# The ReadFree tool for the identification of poor readers: a validation study based on a machine learning approach in monolingual and minority-language children

**DOI:** 10.1007/s11881-023-00287-3

**Published:** 2023-08-07

**Authors:** Desiré Carioti, Natale Adolfo Stucchi, Carlo Toneatto, Marta Franca Masia, Milena Del Monte, Silvia Stefanelli, Simona Travellini, Antonella Marcelli, Marco Tettamanti, Mirta Vernice, Maria Teresa Guasti, Manuela Berlingeri

**Affiliations:** 1 DISTUM, Department of Humanities, University of Urbino Carlo Bo, Urbino, Italy; 2 Psychology Department, University of Milano-Bicocca, Milan, Italy; 3 Center of Developmental Neuropsychology, AST Pesaro-Urbino, Distretto di Pesaro, Pesaro, Italy; 4 Department of Human Sciences, University of the Republic of San Marino, San Marino, Republic of San Marino; 5 NeuroMi, Milan Center for Neuroscience, Milan, Italy

**Keywords:** Bilingualism, Classification and regression tree (CART) model, Developmental dyslexia, Executive functions, Timing skills

## Abstract

In this study, we validated the “*ReadFree tool*”, a computerised battery of 12 visual and auditory tasks developed to identify poor readers also in minority-language children (MLC). We tested the task-specific discriminant power on 142 Italian-monolingual participants (8–13 years old) divided into monolingual poor readers (*N* = 37) and good readers (*N* = 105) according to standardised Italian reading tests. The performances at the discriminant tasks of the *“ReadFree tool*” were entered into a classification and regression tree (CART) model to identify monolingual poor and good readers. The set of classification rules extracted from the CART model were applied to the MLC’s performance and the ensuing classification was compared to the one based on standardised Italian reading tests. According to the CART model, auditory go-no/go (regular), RAN and Entrainment_100bpm_ were the most discriminant tasks. When compared with the clinical classification, the CART model accuracy was 86% for the monolinguals and 76% for the MLC. Executive functions and timing skills turned out to have a relevant role in reading. Results of the CART model on MLC support the idea that ad hoc standardised tasks that go beyond reading are needed.

**Supplementary Information:**

The online version contains supplementary material available at 10.1007/s11881-023-00287-3.

## Introduction

The need for available tests and tools for a fast and accurate screening of reading deficits comes from the high number of children notified by schools and teachers as being at risk of developmental dyslexia and, more in general, of learning disorders. Not all of them receive a fast diagnosis, and once neuropsychologically assessed, not all of them manifest reading disorders. These critical facts add to specific concerns on the neuropsychological assessment of multilingual students, for which there is still a lack of ad hoc created tests and of clinical consensus and criteria, even if literacy difficulties have been documented for first- and second-generation immigrants (Arikan et al., 2017; Bonifacci & Tobia, 2016; Rangvid, 2007; Schnepf, 2004 in Italy by Azzolini et al., 2012; Murineddu et al., 2006). This issue has been recently highlighted in the new “Italian guidelines for the identification of Specific Learning Disorder”, Recommendation 7.3^1^. This recommendation (p. 72) reports that “for the identification of Specific Learning Disorders (i.e., Dyslexia and Dysorthography) in a bilingual population, it is recommended to use tests standardised on a bilingual sample”.

To address this issue, we envisaged a computerised screening tool, namely the *ReadFree tool*, capable of detecting behavioural cognitive markers of reading difficulties in both monolinguals and minority-language children (MLC). Our battery minimises the involvement of language processing to obtain cognitive measures free from potential biases associated with use and exposure to variable languages, such as in the case of MLC. Our final goal is to optimise the assessment requests that specialised neuropsychological centres receive, thus reducing the level of burden sustained by National Health Systems.

### Markers of developmental dyslexia

Developmental dyslexia is a neurodevelopmental disorder “characterised by problems with accurate or fluent written word recognition, poor decoding, and poor spelling abilities” (DSM-5; American Psychiatric Association, 2013, p. 67). DSM-5 (American Psychiatric Association, 2013) describes the specific learning impairment of readers with dyslexia as an “unexpected” inability to acquire literacy despite preserved reasoning skills. Indeed, to be classified as readers with dyslexia, individuals must show reading skills “below age expectations” despite adequate instruction and educational opportunities, and despite the presence of intact non-verbal reasoning skills, intelligence, and sensory abilities (Peterson & Pennington, 2015). Causes of dyslexia were investigated by several theoretical frameworks (see Carioti et al., 2021; Peterson & Pennington, 2012; Stein, 2018 for reviews) and, accordingly, many empirical findings were provided about dyslexia in different cognitive and perceptual domains. For this reason, dyslexia is described by recent accounts as a multiple deficit disorder, in which different patterns of underlying cognitive deficits can characterise the reading impairment (McGrath et al., 2020; Pennington, 2006; Pennington et al., 2012; Ring & Black, 2018). This view that has been supported by comparative studies (Danelli et al., 2017; Ramus et al., 2003; Reid et al., 2007), although the more replicated findings seem to be those concerning the phonological deficit (Bradley & Bryant, 1978; Elbro & Jensen, 2005; Joanisse et al., 2000; Ramus & Szenkovits, 2008).

Indeed, as widely observed in the literature, beyond the obvious differences in reading tasks, readers with dyslexia may show deficits in cognitive dimensions such as phonological awareness, rapid automatized naming (RAN), verbal working memory (see Carioti et al., 2021 for a review), and also non-verbal abilities related to auditory perception (Goswami et al., 2011; Huss et al., 2011; Tallal, 1980; Tallal & Gaab, 2006; Thomson et al., 2006; Thomson & Goswami, 2008) and visuo-attentional skills (Facoetti et al., 2019; Facoetti & Molteni, 2001; Facoetti, Paganoni, & Lorusso, 2000a; Franceschini et al., 2012). Interestingly, some of these cognitive markers of the disorder (phonological awareness, RAN, verbal working memory) are universal and were, thus, found to be impaired in dyslexia across ages and orthographies (see Carioti et al., 2021 for a review).

Above these most evaluated cognitive skills, several non-verbal paradigms were put forward by experimental psychology to verify causal theories of dyslexia such as the rapid auditory processing theory (Tallal, 1980), the perceptual anchoring theory (Banai & Ahissar, 2010), the temporal sampling theory (Goswami et al., 2011; Huss et al., 2011), and the magnocellular theory (Stein & Walsh, 1997). These non-verbal or language-independent paradigms identified some peculiar behavioural alterations in readers with dyslexia also for perceptual skills as, for example, discrimination of tones/phonemes/syllables (Baldeweg et al., 1999; Fostick et al., 2012; Richardson et al., 2004), rapid auditory sequencing (Georgiou et al., 2010; Hari & Kiesilä, 1996; Laasonen et al., 2001), timing skills (Farmer & Klein, 1995; Flaugnacco et al., 2014; Gaab et al., 2007; Tallal & Gaab, 2006; Thomson et al., 2006; Thomson & Goswami, 2008; see Hämäläinen et al., 2013 for a review), motion perception (Hari & Renvall, 2001; Mascheretti et al., 2018; see Benassi et al., 2010 for a review), visuo-attentional skills (Facoetti, Paganoni, Turatto, et al., 2000b), and executive functions tested by means of paradigms such as the Stroop test, the go/no-go task, the Wisconsin card sorting test, and many others (see Booth et al., 2010 and Lonergan et al., 2019 for reviews).

#### Searching for language-independent tasks: rhythm as a matter of phonology

Except for phonological processing and verbal working memory, some of the cognitive skills mentioned above and considered reliable markers of dyslexia do not necessarily involve language processing and can be tested through language-independent tasks. This is very relevant for the issue addressed by the present work, since the identification of dyslexia-related non-verbal deficits offers the opportunity to find cognitive markers of reading deficits also in a population for whom the “linguistic bias” is an obstacle to diagnosis, as in the case of minority-language students.

A growing body of recent works investigated the relationship between rhythmic skills, language, and the reading disorder (Boll-Avetisyan et al., 2020; Flaugnacco et al., 2014; Pagliarini et al., 2020; Thomson et al., 2006; Thomson & Goswami, 2008). Readers with dyslexia seem to show impairments in rhythmic tasks as maintaining a regular tapping both in childhood (Leong & Goswami, 2014a; Thomson & Goswami, 2008) and adulthood (Leong & Goswami, 2014b; Thomson et al., 2006). These findings also promoted rhythmic and musical intervention for dyslexia (Bonacina et al., 2015; Flaugnacco et al., 2015; Overy, 2003; Thomson et al., 2013), often in a computerised form (Cancer et al., 2020). These language-independent auditory tasks may represent an alternative method to assess phonological difficulties that characterize dyslexia. This idea has been supported by studies that explored the relationship between phonological processing and auditory perception (see the rapid auditory processing theory, Gaab et al., 2007; Tallal, 1980; Tallal & Gaab, 2006), detection of stressed metrical elements in language (see the temporal sampling hypothesis, Goswami et al., 2011; Huss et al., 2011), and rhythmic production conceived as a regular planned movement (see the cerebellar theory, Nicolson et al., 2001a, 2001b; Nicolson & Fawcett, 1990, 2011; Nicolson et al., 1999). In other words, the adoption of non-verbal and, thus, language-independent tasks would allow clinicians to assess the children while overcoming the linguistic and orthographic gap that often prevents an unbiased evaluation of bilingual students (Everatt et al., 2000) and MLC.

#### Searching for language-independent tasks: executive functions, RAN, and reading skills

In the same line of reasoning, several studies suggested that reading disorders may be associated with poor executive functions abilities (Barbosa et al., 2019; Brosnan et al., 2002; Doyle et al., 2018; Moura et al., 2014; Reiter et al., 2005; Smith-Spark et al., 2016; Varvara et al., 2014). As mentioned above (paragraph 1.1), several language-independent executive functions tasks were tested on readers with dyslexia (see table 2 in Booth et al., 2010, p. 152); some of them, like the well-known go/no-go task (Donders, 1969, in Gomez et al., 2007), can be easily adapted for clinical and screening tests.

The RAN itself, another reliable marker of dyslexia (Denckla & Rudel, 1976; Wolf & Bowers, 1999; see Araújo & Faísca, 2019 for a review), can be easily transposed in a computerised version that, if realised with a limited number of non-alphanumeric stimuli, would considerably reduce language processing, being suitable also to assess MLC (Carioti et al., 2022).

In line with these considerations, we made an effort to develop a computerised screening tool, i.e., the *ReadFree tool*, capable of identifying both monolingual and MLC at risk of reading disorders without using any reading or linguistic tasks, and also taking into account the auditory-visual dichotomy that seems to characterise different dyslexia profiles (Castles & Coltheart, 1993).

### The multiple deficit model and the need for a multivariate approach

As highlighted by McGrath et al. (2020), the multiple deficit model was proposed as a multilevel framework for understanding neurodevelopmental disorders such as ADHD, autism spectrum disorders, and developmental dyslexia. The passage from a single cognitive deficit, conceived as a core deficit, to the more open idea of a set of cognitive deficits that are probabilistically related to the condition labelled as “dyslexia”, would allow one to better explain the high degree of comorbidities between learning disorders. Moreover, the multiple deficit perspective fits better with the empirical evidence provided in the literature about different profiles of readers with dyslexia, characterised by different cognitive deficits (see Castles & Coltheart, 1993 and the double deficit hypothesis by Wolf & Bowers, 1999). From its first formulation (Pennington, 2006), this multiple deficit account has been tested through several studies that implied multivariate approaches (Moll et al., 2016; Moura et al., 2017; Peterson et al., 2017; Ring & Black, 2018).

The current study attempts to provide a further piece of evidence for supporting the multi-deficit approach and, to this aim, we looked at machine learning as a method that provides a chance to classify children as good and poor readers based on multivariate clinical markers. The adoption of a machine learning–based classification approach has been effective in different disciplines where multivariate clinical markers must be managed: several examples come from biology (see Tarca et al., 2007 and Sommer & Gerlich, 2013 for reviews), medicine (Asri et al., 2016; Ghiasi et al., 2020; Hathaway et al., 2019; Mir & Dhage, 2018), as well as psychology and neuropsychology (e.g., Omar et al., 2019; Rostami et al., 2020; see Battista et al., 2020 and Dwyer et al., 2018 for reviews).

### The classification and regression tree (CART) model

CART models are a machine learning technique of modelling to divide and, thus, classify data based on the recursive partition of a given “training” dataset’s feature space (see Myles et al., 2004 for a review). The method, based on multivariate non-parametric correlations, finds a set of decision rules in which input variables are split in root nodes, based on their information gain. Accordingly, the decision tree is built based on the set of sequential rules that better replicate the classification in input. Based on this first training, the CART extracts a series of predictions, i.e., the decision tree model, that can be applied to a new dataset, usually known as the “testing” one (Myles et al., 2004; Pradhan, 2013; Rostami et al., 2020).

This classification approach needs enough variables in input and, thus, is by definition suited to handle a multivariate set of data. The fact itself of obtaining a set of classification rules makes the CART approach a relevant technique to adopt for diagnostic purposes. Moreover, the hierarchical variables’ structure emerging from the tree is highly informative. Indeed, thinking about neuropsychological multiple deficit disorders such as ADHD, autism spectrum, and developmental dyslexia, the use of CART models provides a wide range of advantages (Omar et al., 2019; Rostami et al., 2020). From a diagnostic point of view, these models provide an automated way for classifying participants while also allowing to better understand the role of specific deficits and their reciprocal links. In our study, this approach would provide the opportunity to better understand what language-independent deficits prevail in dyslexia and, based on the number of tree branches, whether we can empirically observe different behavioural profiles. In other words, the adoption of such a multivariate approach allows us to advance in the knowledge of this complex deficit from a comparative perspective, thus supporting from a novel angle the multiple deficit approach proposed by Pennington (2006). Moreover, in line with one of the goals of this work, CART will allow us to apply the decision rules based on several language-independent tasks to MCL, that is to a group for which standardised clinical tests are not yet available.

### Aim of the present study

In this study, we aim at validating our *ReadFree* tool by testing criterion validity of the whole screening battery, i.e., a component of construct validity (based on Anastasi, 1986; Messick, 1979) that represent the degree to which a test can predictively (in the future) or concurrently (in the present) measure something concerning a specific hypothesis (criterion) and, thus, latent construct. To do so, we (*step 1*) first tested the task-by-task criterion validity by testing whether each ReadFree task can be associated with good and poor reading performances. Children were previously identified as good or poor readers by adopting standardised Italian reading tests and clinical criteria (see paragraph 2.1 for a detailed description), i.e., by adopting a set of tasks independent of our *ReadFree* tool to avoid circularity.

Once identified and selected the discriminant tasks, we (*step 2*) explored the between-task patterns of correlations and the nature of their relationship with reading and reading-related cognitive skills. This was done to check whether the ReadFree tasks cover different cognitive-related aspects of the reading process, as they were supposed to do when we developed the screening. In other words, we checked whether the outcomes of our screening can be directly related to reading proficiency, for further testing the validity argument known as “explanation inference”. Indeed, explanation inference is about understanding the theoretical relationship between the test content and the construct of interest, so, in our case, the relationships between ReadFree tasks and the reading process (see Chapelle et al., 2008; Knoch & Chapelle, 2018). This second step was realised through a principal component analysis that let us explore also potential latent factors underlying reading and language-independent cognitive skills. This procedure has been applied only to monolingual good readers to deepen our understanding of aspects related to the typical reading process.

As a third step (*step 3*), we further tested the criterion validity of the whole *ReadFree* tool after excluding non-discriminant tasks. In this step, we adopted a recursive-partitioning machine learning approach based on a classification and regression tree (CART; see paragraph 1.3.1) model to extract a multivariate set of classification rules for discriminating between good and poor monolingual readers. While steps 1 to 3 were focused on the Italian-monolingual sample, in *step 4*, we compared MLC’s performances with those of Italian monolinguals (both good and poor readers) at standardised reading tasks and tasks of the *ReadFree* tool. This step was necessary to check whether we can apply the same reasoning used for monolinguals also to the MLC population. Therefore, we assume that tests capable of identifying poor readers in a population (monolinguals) will be capable of identifying them also in another population with a similar distribution of good and poor readers (MLC), but we have to prove that the two populations have the same reading behaviour and same performances in the ReadFree tasks. This can be considered as a preliminary step to then test the external validity (Campbell, 1957; Ferguson, 2004; see Findley et al., 2021 for a review) of our instrument. In the last step (*step 5*), the classification rules extracted in *step 3* from the sample of monolinguals using the CART model were applied to MLC for identifying poor and good readers in this other group. Once obtained an MLC classification based on the CART predictions, this was compared to the one we obtained by standardised clinical reading tests to compute our tool’s performance measures. Results of these indices are, anyway, to interpret with caution since standardised clinical tests cannot be considered “gold standard” tests for comparison, as we will better explain later in the text. These performance measures will also provide a “reliability” measure of the ReadFree classification rules, as they will represent the goodness of fit of these rules when applied to other participants. Accordingly, with this last step, we will test the generalizability and, thus, the external validity of our tool conceived as the “extent to which inferences drawn from a given study’s sample apply to a broader population or other target populations” (Findley et al., 2021, p. 366).

This multistage validation process (see Anastasi, 1986 for a review) aims at obtaining a final version of the *ReadFree tool* that will be further validated on a larger sample in further studies.

## Materials and methods

### Participants

A total number of 257 primary and middle school students were involved in the data collection. After having excluded dropouts (*n* = 5), participants with incomplete data on more than two tasks (*n* = 3), participants who had lived for less than a year in Italy, and, as a consequence, were not enough proficient in Italian (*n* = 7), and children with other neurodevelopmental disorders beyond reading difficulties (*n* = 14), we obtained a sample of 228 participants. Of these, 46 were 3rd graders (girls = 24, boys = 22; age in moths, mean = 103.83, SD = 4.26), 49 were 4th graders (girls = 22, boys = 27; age in months, mean = 114.94, SD = 4.64), 41 were 5th graders (girls = 29; boys = 12; age in months, mean = 127.8, SD = 3.54), 30 were 6th graders (girls = 15, boys = 15; age in months, mean = 141.2, SD = 3.68), 28 were 7th graders (girls = 12, boys = 16; age in months, mean = 153.82, SD = 5.29), and 34 were 8th graders (girls = 12, boys = 22; age in months, mean = 161.79, SD = 5.53).

Students were included in 3 groups based on their parents’ nationality and their performances on reading tests. Accordingly, we obtained (i) a control group of monolingual good readers (GR; *n* = 105) with both Italian parents and without reading disorders; (ii) a group of monolingual students that were evaluated as poor readers (PR; *n* = 37) based on standard clinical reading and cognitive tests; and (iii) a group of minority-language children (MLC; *n* = 68) with one or both foreign parents and a bilingual linguistic family context.

MLC students were heterogeneous for both language of origin and minority-language exposure. The degree of cumulative exposure for each MLC has been investigated using the PLQ Interview (Intervista delle Prassi Linguistiche Quotidiane - Daily Linguistic Practice Interview by Carioti et al., 2022a, b, September 13), a structured interview conceived for assessing language use and experience on both the minority and the majority language on MLC. The amount of time spent speaking the minority language with the mother (average percentage time in a day = 11.23%, SD =11.7) or father (average percentage time in a day = 5.85%, SD = 6.5) was very heterogeneous and several children (*n* = 21) declared that they did not speak the minority language at home. However, all of them were daily (passively) exposed to the minority language in the family context for at least half an hour (average percentage time in a day = 18.4%, SD = 10.12). Parents’ languages of origin, reported in Supplementary Table 1, were very heterogeneous too. All MLC had Italian as the main language of education.

Accordingly, we further excluded children (*n* = 18) with low performance in non-verbal reasoning, i.e., a Raven’s matrices’ score below the 50th percentile (see paragraph 2.2 for details about the standardised test). Consequently, we obtained a final sample of 210 participants aged between 8 and 13 years old. Demographic information and non-verbal reasoning scores of the three groups are reported in Table 1.

**Table 1:** Demographic data and non-verbal reasoning average scores

	Years	Gender		Handedness	*N* total	Age (months)		Raven’s average score (percentile)	
		Girls	Boys	(R/L)		Mean	SD	Mean	SD
*GR*	8	8	8	16/0	16	102.25	3.51	94.94	4.22
	9	12	13	24/1	25	113.48	3.45	95.08	4.71
	10	15	6	20/1	21	126.9	3.19	94.48	8.57
	11	6	8	14/0	14	139	3.53	80.86	10.63
	12	7	6	12/1	13	150	4.6	83.62	16.03
	13	6	10	16/0	16	161	3.12	86.94	10.13
	Tot.	54	51		105			90.53	10.66
*PR*	8	2	0	1/1	2	103.5	2.12	94	8.49
	9	6	5	11/0	11	112.82	4.21	83.09	14.78
	10	2	3	4/1	5	124.4	2.97	91.4	15.95
	11	2	3	4/1	5	139.8	4.49	79	10.15
	12	2	5	6/1	7	151.29	1.89	85	18.37
	13	2	5	6/1	7	158.86	2.79	75.86	19.09
	Tot.	16	21		37			83.24	15.77
*MLC*	8	7	9	15/1	16	102.5	3.06	94.96	6.59
	9	7	11	17/1	18	111.94	3.61	89.39	12.79
	10	9	5	12/2	14	126	3.55	87.58	12.58
	11	6	2	7/1	8	135.62	3.2	92.5	9.62
	12	1	1	2/0	2	154	1.41	86	8.49
	13	5	5	9/1	10	160.6	3.96	80.1	16.67
	Tot.	35	33		68			89.16	12.32

All participants were enrolled in primary and middle public schools in Northern and Central Italy. Data of primary students were collected in the “I.C. Della Torre” of Chiavari (Genova), while data of middle school students were collected in the “I.C. Lanfranco" of Gabicce Mare and Gradara (Pesaro-Urbino). Some other monolingual (15/142 = 10.5%) and MLC (7/68 = 10.2%) participants were enrolled in the Center of Developmental Neuropsychology, AST Pesaro-Urbino. Twenty-two students of the monolingual PR group had an official diagnosis of developmental dyslexia (19 in the Center of Developmental Neuropsychology and 3 by a professional neuropsychologist elsewhere). Five more students tested in school were included in the monolingual PR due to their poor reading performances.

None of these children had psychiatric, emotional, or sensory disabilities, and all participants had normal or corrected-to-normal visual acuity. According to the World Medical Association Declaration of Helsinki’s ethical principles, informed consent was obtained from parents, and children gave their verbal consent to participate in the study. The Ethical Committee of the University of Urbino Carlo Bo approved the study (prot. Num. 11, 20^th^ August 2018). Some participants (i.e., primary students) were also included in the sample of one of our previous studies (Carioti et al., 2022a, b).

### Cognitive assessment

Participants were assessed with the following neuropsychological battery, including standardised clinical reading tests:

1. Raven’s coloured progressive matrices (CPM; Raven, 1956) and standard progressive matrices (SPM; Raven, 2003; Raven, 1958), that is a set of pattern-matching tasks in which participants must determine the final pattern in a series. This task was used to assess non-verbal reasoning.
2. The digit forward and backward subtests of the WISC-IV (Wechsler, 2003), that is a set of tasks that require children to retain in memory a series of digits (forward version) or to retain and reverse the digits’ order (backward). These subtests assess both short-term and working memory. Here, the raw score corresponded to the maximum number of digits recalled (i.e., the memory span).
3. Nonwords repetition test from the VAUMeLF battery (Batterie per la Valutazione dell'Attenzione Uditiva e della Memoria di Lavoro Fonologica nell'Età Evolutiva; Bertelli & Bilancia, 2008), a task in which 40 nonwords delivered by a recorded voice must be repeated by children. The test assesses auditory attention and phonological skills. If an item was correctly repeated after the first listening, then the child obtained a score of 1; if the nonword was repeated at the second listening, the score was 0.5. The raw score corresponded to the sum of the scores obtained for each item.
4. Single word and pseudoword reading was assessed through the DDE-2 test (Batteria per la Valutazione della Dislessia e della Disortografia Evolutiva-2; Sartori et al., 2007), which requires children to read a series of words/pseudowords presented in lists. The word reading test assesses whole-word decoding and, thus, lexical identification, while the pseudoword reading test assesses the phonological decoding skills, i.e., the grapheme-to-phoneme mapping.
5. Text reading was assessed using the short-stories in the battery MT and MT-3 Clinica (Cornoldi & Caretti, 2016; Cornoldi & Colpo, 2002).

For each of the 3 reading tasks (words—pseudowords—text), we obtained a fluency (syllables/seconds) and an accuracy score (percentage of accuracy), for a total of 6 measures of reading proficiency.

### Experimental tasks

The battery consisted of 12 tasks organised with a hierarchical logic. Half of the tasks were realised in the auditory and the other half in the visual modality to obtain parallel correspondent tasks for both channels (see Table 2 for a summary of the tasks included in the *ReadFree tool*, with detailed description of experimental phases, number of trials for each phase, variants of the task, and the scoring procedure). Only two tasks did not have a correspondent counterpart: the RAN-shapes in the visual modality and the cocktail party effect task in the auditory one. While the first task (the RAN), preceded by a training trial of single naming, was included to test the speed of retrieving a selected pool of lexical labels, the second one (the cocktail party effect task) assessed selective auditory attention. Both were designed to test, even if in a different format, selective attention and to stress “the crowding effect” (see Gori & Facoetti, 2015). Behavioural tasks included in the screening tool are summarised in Table 2. An extended description of each task is included in the supplementary materials.

**Table 2:** Tasks included in the ReadFree tool

ReadFree tool									
Auditory modality					Visual modality				
	*Task*	*N trial*	*Variants*	*Scores*		*Task*	*N trials*	*Variants*	*Scores*
*Reaction time*	The participant must click the mouse as soon as a sound is delivered	10		Median RTs of 8 trials (the first two trials are not considered)	*Reaction time*	The participant must click the mouse as soon as a dot appears on the screen	10		Median RTs of 8 trials (The first two trials are not considered)
*Tapping*	Listening: the participant must listen to a rhythmic pulse delivered by a metronome	16	80 bpm 100 bpm		*Tapping*	Listening: the participant must watch a dot regularly presented on the screen	16	80 bpm 100 bpm	
	Entrainment: the participant must click the mouse aligned to the metronome	16		Tapping onset = degree of anticipation or delay with respect to the target stimulus		Entrainment: the participant must click the mouse aligned to the dot	16		Tapping onset = degree of anticipation or delay with respect to the target stimulus
	Free tapping: the participant must reproduce the rhythm without listening to any stimulus	12				Free Tapping: the participant must reproduce the rhythm without watching any stimulus	12		
*Tone discrimination*	The participant must indicate on the keyboard whether the pair of tones presented are “same” or “different”	Adaptive procedure based on Viviani and Stucchi (1989)		Degree of discriminant sensitivity - smallest interval perceived	*Grey-scale discrimination*	The participant must indicate on the keyboard whether the pair of grey squares presented are “same” or “different”	Adaptive procedure based on Viviani and Stucchi (1989)		Degree of discriminant sensitivity - smallest interval perceived
*Cocktail party*	The participant must find a target tone in the noise. The noise is represented by distractors tones of different timbres and pitch in the range of human voice.	36.5 s of stimulation × 3 trials	-3 voices noise -10 voices noise -5 voices noise	d-prime	*RAN-shapes*	The participant must rapidly name all the shapes presented in a grid	3	– 7x7 simple matrix - 10x10 matrix - 7x7 matrix with visual interference See Carioti et al. (2022a, b)	Shapes named in 30 seconds
*Auditory go/no-go*	The participant is instructed to click the mouse when a low tone is heard and not to click when a higher one is heard	2	Irregular: random ISI Regular: ISI at 60 bpm	d-prime	*Visual go/no-go*	The participant is instructed to click the mouse when a grey dot appears and not to click when a yellow one appears	2	Irregular: random ISI Regular: ISI at 60 bpm	d-prime
*Auditory anticipatory timing*	The participant must click the mouse when the second tone of a pair is delivered, aligned with a regular timing	1	Pagliarini et al., 2020	Tapping onset = degree of anticipation or delay compared to the target stimulus	*Visual anticipatory timing*	The participant must click the mouse when the second big dot of a pair is delivered, aligned with the regular presentation of smaller dots.	1		Tapping onset = degree of anticipation or delay compared to the target stimulus

### Experimental procedures

Children were tested individually in two different sessions: in the first one, participants underwent the cognitive battery, and in the second one the *ReadFree* tool was administered.

All the tasks included in the *ReadFree* tool were developed in the Matlab environment (2018b, www.MathWorks.com) and presented through a PC DELL Inspiron 15 5000, with a 15.6 inches screen, Intel Core™ i7-1165G7 driver, and Windows Home 10 Operative System. Each participant was set in front of the PC and asked to wear headphones (Philips Bass + SHL3075WT/00 with integrated microphone) to listen to the battery’s auditory tasks and to provide the vocal answers required in the RAN-shapes task. A Logitech M110 silent mouse was used for participants’ responses to avoid any conflicting sound during auditory tasks. Instructions about each task were orally provided by the researchers and some training sessions were included in many tasks (cocktail party, go/no-go, warning imperative). Participants were allowed to ask the researchers for more information before each task started and/or to repeat the training session. No instructions were delivered through text to avoid further complications for poor readers.

### Data analyses

All the analyses were performed in the R environment (R Core Team, 2019). Different approaches, implying both unidimensional and multidimensional analyses, were used to test each task’s discriminant power, as well as the validity of the *ReadFree* tool.

#### Step 1

First of all, we explored data distributions through boxplots to detect eventual outliers (i.e., data < 1^st^ or > 3^rd^ quartile + 1.5 IQR). After this check, we used logit models to test the discriminant power of every task; age was included in each model as a covariate. This first step allowed us to reduce the number of tasks involved in the battery and select a set of variables to be included in the multivariate analysis. This technique is commonly used to avoid large standard errors and the risk of identifying spurious associations (Ranganathan et al., 2017). As explained above, this step let us test each task’s criterion validity.

#### Step 2

Performances of monolingual good readers at experimental tasks, reading, and reading-related cognitive skills were included in a PCA. This approach has already been used in several studies to assess the construct validity (particularly, criterion validity) of neuropsychological batteries (Baser & Ruff, 1987; Shum et al., 1990; Vogel et al., 2015), also in the case of computerised tools (Berger et al., 1997; Kabat et al., 2001; Smith et al., 2013). This choice was made because it gave us the chance to explore the pattern of correlations between measures in complex cases, as the present one, and to better explore the relationship between single tasks and specific aspects of a high-order cognitive process underlying accurate and fast reading processing. The analysis was performed using the *principal* function of the “psych” R package (Revelle, 2014) and the *prcomp* function of the “stats” R package. As we assumed a high rate of between-variable correlations, in line with one of our previous studies (Carioti et al., 2019), we applied an Oblimin rotation to factors extracted based on the scree-plot exploration.

#### Step 3

We adopted a machine learning technique to obtain a hierarchical classification of the discriminant tasks (see paragraph 1.3.1). Accordingly, monolinguals GR and PR data were used to train a classification and regression tree (CART) model (Breiman et al., 1984), by using the *rpart* R package (Therneau et al., 2015). As the classification of GR and PR based on standardised clinical tests was reliable only for Italian monolinguals, only their data were included in the *training dataset*. The number of root nodes included in the decision tree was decided in relation to model’s complexity: the tree was cut at the last variable which reduced the complexity. The classification made by the CART model has been compared to the one in input for extracting the ReadFree tool’s performance measures. In particular, the performance measures considered were (i) sensitivity, i.e., the number of true positives (TP) on all positive assessments; (ii) specificity, i.e., the number of true negatives (TN) on all true negative assessments; (iii) positive predictive value, i.e., the proportion of positive test results in the group of actual poor readers [TP/(TP + FP)]; the negative predictive value, i.e., the proportion of participants that resulted negative for the condition on actual good readers [TN/(TN + FN)]; and (iii) the overall accuracy (see Eusebi, 2013; Glaros & Kline, 1988; Trevethan, 2017). The overall accuracy of the instrument, thus calculated as the proportion of true positive (TP) and true negatives (TN) on the entire sample [(TN + TP)/(TN + TP + FN + FP)] (Berlingeri et al., 2019; Šimundić, 2009), as the above-mentioned performance indices, will be presented in terms of percentage of participants in which the CART classification corresponds to the one made through standardised clinical reading tests.

#### Step 4

Performances of Italian monolinguals with and without reading difficulties (monolingual GR + PR) were compared to those of MLC in standardised reading tasks as well as in the discriminant tasks of our *ReadFree* tool, using generalised linear models (GLMs). The group was included into GLMs as a fixed factor and the age (in years) as a covariate. When data did not fit the normal distribution, data transformation and alternative family distributions were applied. When this was not possible, a robust non-parametric model was run, using the *lmrob* of the “robustbase” R package (Maechler et al., 2020) or, in case of censored data, a Tobit regression model was applied (Long, 1997; McDonald & Moffitt, 1980) through the R package VGAM (Yee, 2008). The influence of the socio-economic-status (SES)^2^ on the performance at reading and ReadFree tool’s tasks was preliminary checked using the intraclass correlation coefficient (ICC; ICC package— Wolak, 2015) (see Supplementary Table 3). This check let us include in our GLMs only variables that significantly contributed to explaining variance.

As previously mentioned, in *step 4* we wanted to test whether our *ReadFree* tool could be considered empirically suitable for both monolinguals and MLC, assuming that the “language-independent” and “reading free” nature of our tasks make them effective in identifying poor and good readers in MLC as well as in monolinguals. As previously mentioned (paragraph 1.4), starting from the assumption that the two populations had the same good and poor readers distribution, we would not expect any between-group differences in reading or in the experimental tasks and, accordingly, we could safely assume that the *ReadFree* tool could be employed with MLC for identifying “poor readers”, as it can be with monolinguals. In line with this reasoning, we used generalised linear models (GLMs) to test between-group differences. Here, it is noteworthy that, although between-group differences at every task were tested with a dedicated GLM, the p-values of all GLMs were corrected for false discovery rate (FDR) using the procedure of Benjamini and Yekutieli (2001) to control for multiple comparisons. This step supports our further aim of including MLC in the “testing dataset” of the CART model, extending the prediction obtained from the “training dataset” of monolinguals to a different dataset that can be safely considered as an independent subset of the general dataset.

#### Step 5

As a final step, the set of rules extracted by the decision tree on Italian monolingual readers (i.e., “training dataset”, see *step 3*) were applied to the data of the MLC group. MLC participants were included in the “Testing dataset” (32.3% of the total sample) and classified by the CART algorithm as poor readers (MLC-PR) or good readers (MLC-GR). The classification of MLC extracted by the CART model was finally compared to the one obtained by applying clinical reading tests to get the ReadFree tool’s performance measures presented above. Here again, it is worthy to remember that the normative data of the clinical tests included in the neuropsychological assessment were all collected in Italian-monolingual students. This is something that is not recommended from either a methodological or clinical point of view (see Recommendation 7.3 of the “Italian Guidelines for the Identification of Specific Learning Disorder”). However, the standardised clinical tests were the only set of measures that we could use as reference point to test the performance measures of our *ReadFree* tool.

### Results

#### Step 1. Discriminant power of experimental tasks

The results of the logistic regressions are reported in Table 3. These analyses were run to identify the set of ReadFree tool’s tasks capable of discriminating between monolingual GR and PR.

**Table 3:** Results of the logit model run on each task of the ReadFree tool

	GR (*N*)	PR (*N*)	Participants (*N*)	Effect	*X*^2^	DF	*p*-value
*Auditory reaction time*	105	36ˆ	141	Task	1.34	1	0.24
				Age	2.81	1	0.09
				Task*Age	0.17	1	0.67
***Visual reaction time***	101ˆ	35ˆ	136	Task	4.95	1	0.02*
				Age	4.94	1	0.02*
				Task*Age	0.03	1	0.85
*Auditory entrainment*_*80bpm*_	104^#^	37	141	Task	1.05	1	0.3
				Age	1.39	1	0.23
				Task*Age	0.005	1	0.94
***Auditory entrainment***_***100bpm***_	103^#^ˆ	37	140	Task	4.02	1	0.04*
				Age	3.77	1	0.28
				Task*Age	1.15	1	0.05*
*Auditory free tapping*_*80bpm*_	104^#^	37	141	Task	6.95	1	.008**
				Age	2.38	1	0.12
				Task*Age	0.08	1	0.76
***Auditory free tapping***_***100bpm***_	103^#^ˆ	37	140	Task	6.54	1	0.01*
				Age	0.45	1	0.5
				Task*Age	1.36	1	0.24
*Visual entrainment*_*80bpm*_	105	37	142	Task	1.06	1	0.3
				Age	1.76	1	0.18
				Task*Age	2.81	1	0.09
*Visual entrainment*_*100bpm*_	104ˆ	37	141	Task	3.43	1	0.09
				Age	1.29	1	0.22
				Task*Age	2.01	1	0.11
*Visual free tapping*_*80bpm*_	105	37	142	Task	1.55	1	0.21
				Age	1.68	1	0.19
				Task*Age	1.55	1	0.21
*Visual free tapping*_*100bpm*_	104ˆ	37	141	Task	3.35	1	0.07
				Age	1.96	1	0.16
				Task*Age	1.87	1	0.17
*Tone discrimination*	104^^^	34^^^	138	Task	0.28	1	0.59
				Age	0.76	1	0.38
				Task*Age	1.81	1	0.17
*Grey-scale discrimination*	105	37	142	Task	0.46	1	0.49
				Age	1.76	1	0.18
				Task*Age	0.35	1	0.55
***Cocktail party task***	100^#^^	35^#^^	135	Task	4.43	1	0.05*
				Age	3.56	1	0.11
				Task*Age	3.08	1	0.02*
***RAN-shapes***	105	37	142	Task	26.38	1	<0.001***
				Age	17.15	1	<0.001***
				Task*Age	0.55	1	0.45
***Auditory go/no-go irregular***	104ˆ	37	141	Task	3.65	1	<0.001***
				Age	6.04	1	0.013*
				Task*Age	0.00	1	0.99
***Auditory go/no-go regular***	105	37	142	Task	22.71	1	<0.001***
				Age	7.21	1	0.007**
				Task*Age	3.18	1	0.07
***Visual go/no-go irregular***	99^#^^	36^^^	135	Task	5.38	1	0.02*
				Age	2.42	1	0.11
				Task*Age	1.41	1	0.23
***Visual go/no-go regular***	104^#^	37	141	Task	13.28	1	<0.001***
				Age	2.05	1	0.15
				Task*Age	0.001	1	0.97
*Auditory anticipatory timing*	105	37	142	Task	1.08	1	0.29
				Age	1.61	1	0.2
				Task*Age	1.18	1	0.2
*Visual anticipatory timing*	102^#^	37	139	Task	1.92	1	0.16
				Age	1.23	1	0.26
				Task*Age	0.02	1	0.86

***p < .001, **p < .01, *p < .05

Discriminant tasks are highlighted in bold

^^^Some outliers were removed

^#^Missing data

#### Step 2. Principal component analyses on monolingual GR’s performances

Data of 100 monolingual good readers were included in the PCA. Five participants of the GR group were removed due to missing scores in one ReadFree tool’s task.

**Fig. 1:**
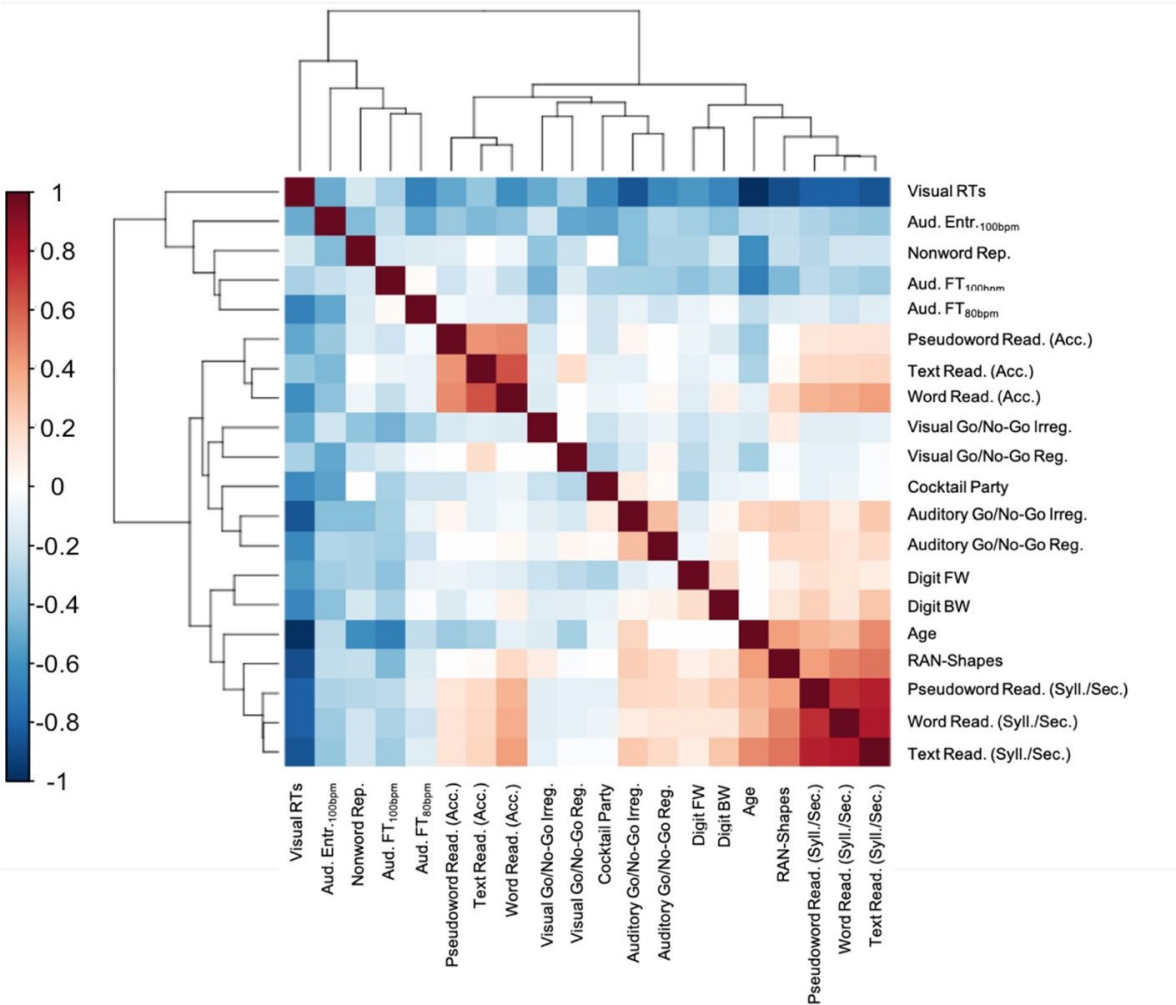
Heatmap and correlational patterns on monolingual good readers. Negative correlations are depicted in blue, while positive ones in red

For each participant, we included in the PCA age (in years), reading and reading-related cognitive tests, and discriminant tasks of the *ReadFree* tool. Patterns of correlations across variables are represented in the heatmap (Fig. 1; the heatmap represents the correlational patterns between reading, cognitive measures, and tasks of the *ReadFree* tool in the sample of monolingual good readers).

**Fig. 2:**
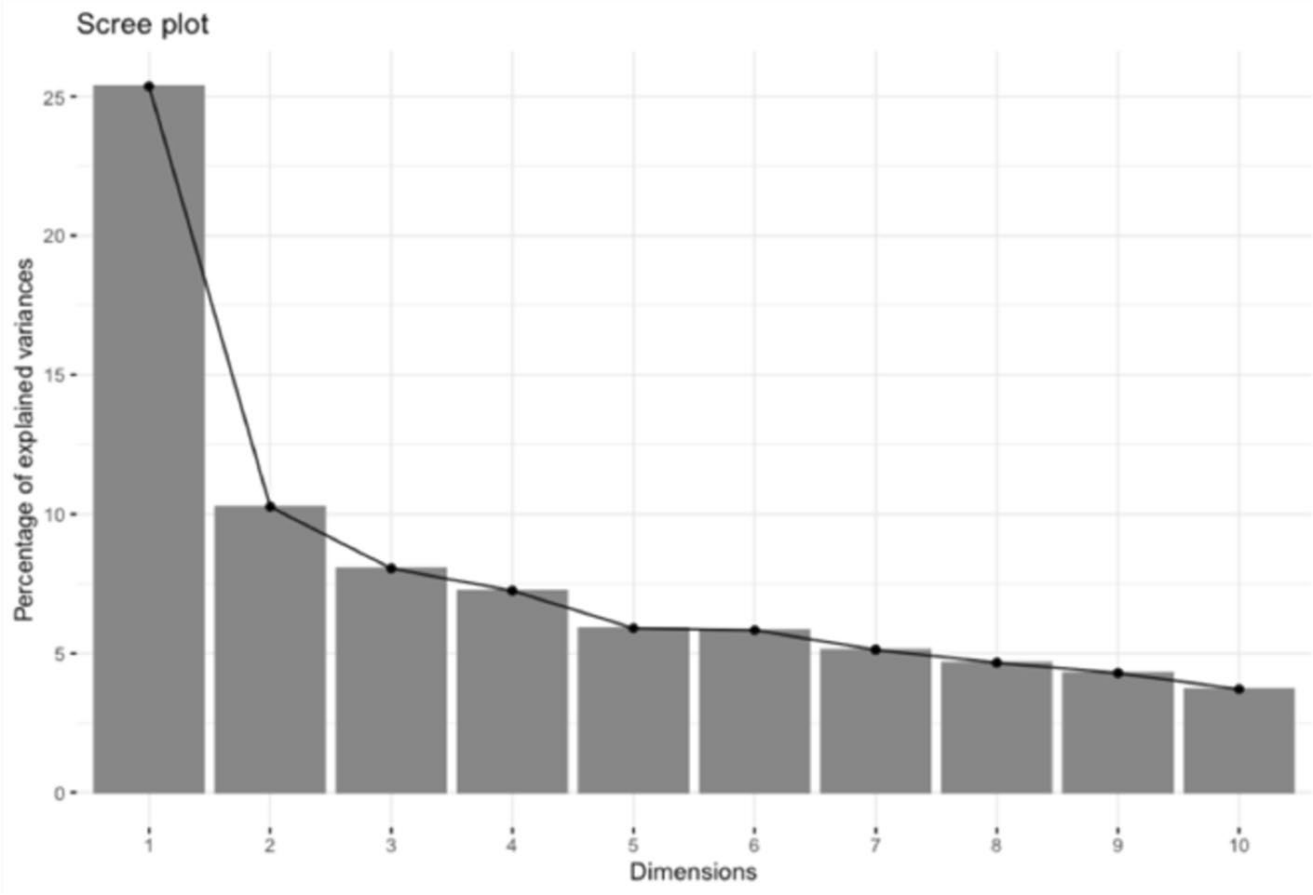
Scree-plot

Based on the scree-plot (see Fig. 2), 4 factors were extracted by the PCA explaining 51% of the variance. Factor loadings produced by the PCA are reported in Table 4 together with commonalities and uniqueness for each variable. Variables with a saturation value > |0.3| were considered to interpret the factors (variables with saturations > |0.5| are reported in bold type).

**Table 4:** Saturation matrix

	F1	F2	F3	F4	Commonalities	Uniqueness
*Text reading (syll./sec.)*	**0.89**	0.09	0.1	0.02	0.83	0.17
*Word reading (syll./sec.)*	**0.85**	0.04	−0.01	−0.12	0.68	0.32
*Pseudoword reading (syll./sec.)*	**0.84**	0.13	0.09	0.01	0.76	0.24
*Age (years)*	**0.79**	−0.13	−0.16	0.18	0.77	0.23
*RAN-shape*	**0.62**	−0.1	−0.13	0.28	0.58	0.42
*Digit FW span*	0.43	0.13	0.19	−0.03	0.24	0.76
*Digit BW span*	0.42	0.03	0.27	0.24	0.38	0.62
*Text reading (% Acc.)*	0.09	**0.79**	−0.07	0.01	0.65	0.35
*Word reading (% Acc.)*	0.44	**0.63**	−0.07	−0.05	0.61	0.39
*Pseudoword reading (% Acc.)*	−0.04	**0.59**	0.19	0.08	0.4	0.6
*Visual Rts*	−0.49	**0.56**	0.04	0	0.52	0.48
*Auditory free tapping*_*80bpm*_	0.24	−0.03	**0.72**	0.01	0.57	0.43
*Auditory free tapping*_*100bpm*_	−0.11	−0.05	**0.66**	0.02	0.44	0.56
*Nonword repetition*	−0.22	0.14	**0.52**	0.12	0.34	0.66
*Auditory entrainment*_*100bpm*_	0.07	−0.17	−0.21	0.19	0.12	0.88
*Auditory go-nogo (regular)*	0.04	−0.07	−0.02	**0.74**	0.57	0.43
*Auditory go-nogo (irregular)*	0.3	−0.14	0.13	**0.57**	0.55	0.45
*Visual go-nogo (irregular)*	0.01	0.25	−0.38	**0.54**	0.5	0.5
*Visual go-nogo (regular)*	−0.12	0.22	0.16	**0.53**	0.35	0.65
*Cocktail party*	−0.04	0.04	0.24	**0.51**	0.31	0.69

As clearly emerged from commonalities (see the saturation matrix with loadings, commonalities, and uniqueness in Table 4), the auditory entrainment_*100bpm*_ and the digit forward had a low contribution to the factorial structure, while the other tasks seemed to be well associated to a specific component of the reading process.

In particular, the first factor (F1) was mainly associated with age, working memory, reading fluency, and RAN (saturation > |0.5|). The second factor (F2) mostly represented reading accuracy, which was associated with visual RTs (saturation > |0.5|). The third factor (F3) isolated phonological awareness and the entrainment and free tapping, i.e., rhythmic auditory tasks (saturation > |0.3|). Lastly, the fourth factor (F4) highlighted the link between the selective auditory attention (cocktail party) and the executive component of inhibition (go/no-go) in both the modalities but did not include any reading or cognitive measures (saturation > |0.5|).

#### Step 3. Classification of good and poor readers based on the CART model

**Fig. 3:**
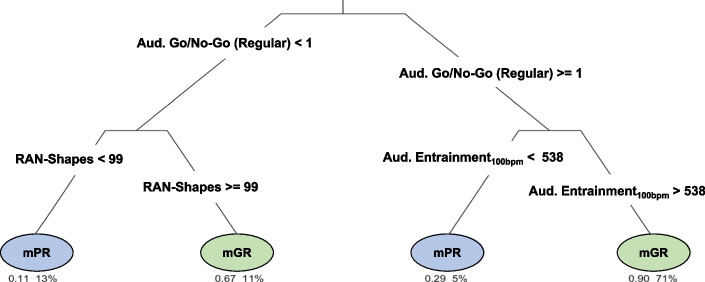
Root nodes and the classification yielded by the CART model

The set of multivariate classification rules is represented in the Decision tree (Fig. 3 and Tables 5–6). Due to some missing data, the CART model was run on the data of 138 participants (102 monolingual GR, 36 monolingual PR). All the discriminant tasks of the *ReadFree* tool, selected with previous univariate analyses, were included as inputs, and the order of root nodes emerged as a function of the information gain score of each task.

**Table 5:** Classification rules yielded by the decision tree

	Classification Rules	
	Left son	Right son
*Auditory go/no-go (regular)*	<1 = PR	≥1 = GR
*RAN-shapes*	<99 = PR	≥99 = GR
*Auditory entrainement*_*100bpm*_	<538 = PR	>538 = GR

**Table 6:** Complexity parameters associated to the CART model

Nodes	cp	*n* splits	Relative error	*x* error	*x* standard
*Auditory go/no-go (regular)*	0.27	0	1	1	0.14
*RAN-shapes*	0.13	1	0.72	1.02	0.14
*Auditory entrainement*_*100bpm*_	0.08	2	0.59	0.89	0.13
*Auditory free tapping*_*100bpm*_	0.01	3	0.51	0.97	0.14

Variables identified as root nodes and splitting rules are reported in Table 5, while complexity parameters (cp) of the model are reported in Table 6.

**Fig. 4:**
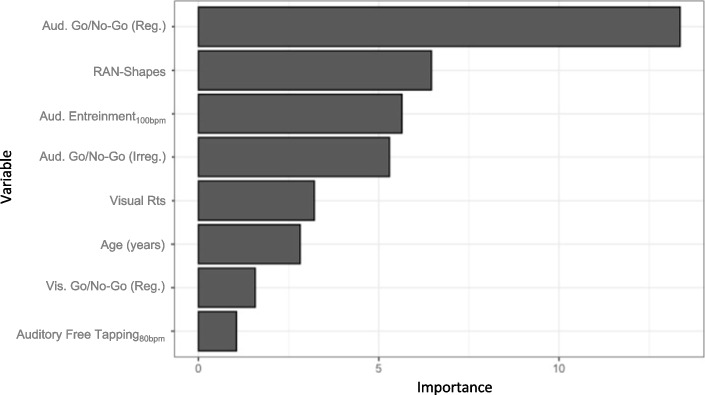
Variable importance order based on the CART model

Variable importance order based on the CART model has been reported in Fig. 4. Each variable’s importance value is computed as the sum of the decrease in impurity. This measure is a function of the variable’s role in both “primary splits” and “surrogate splits” (Breiman et al., 1984).

**Fig. 5:**
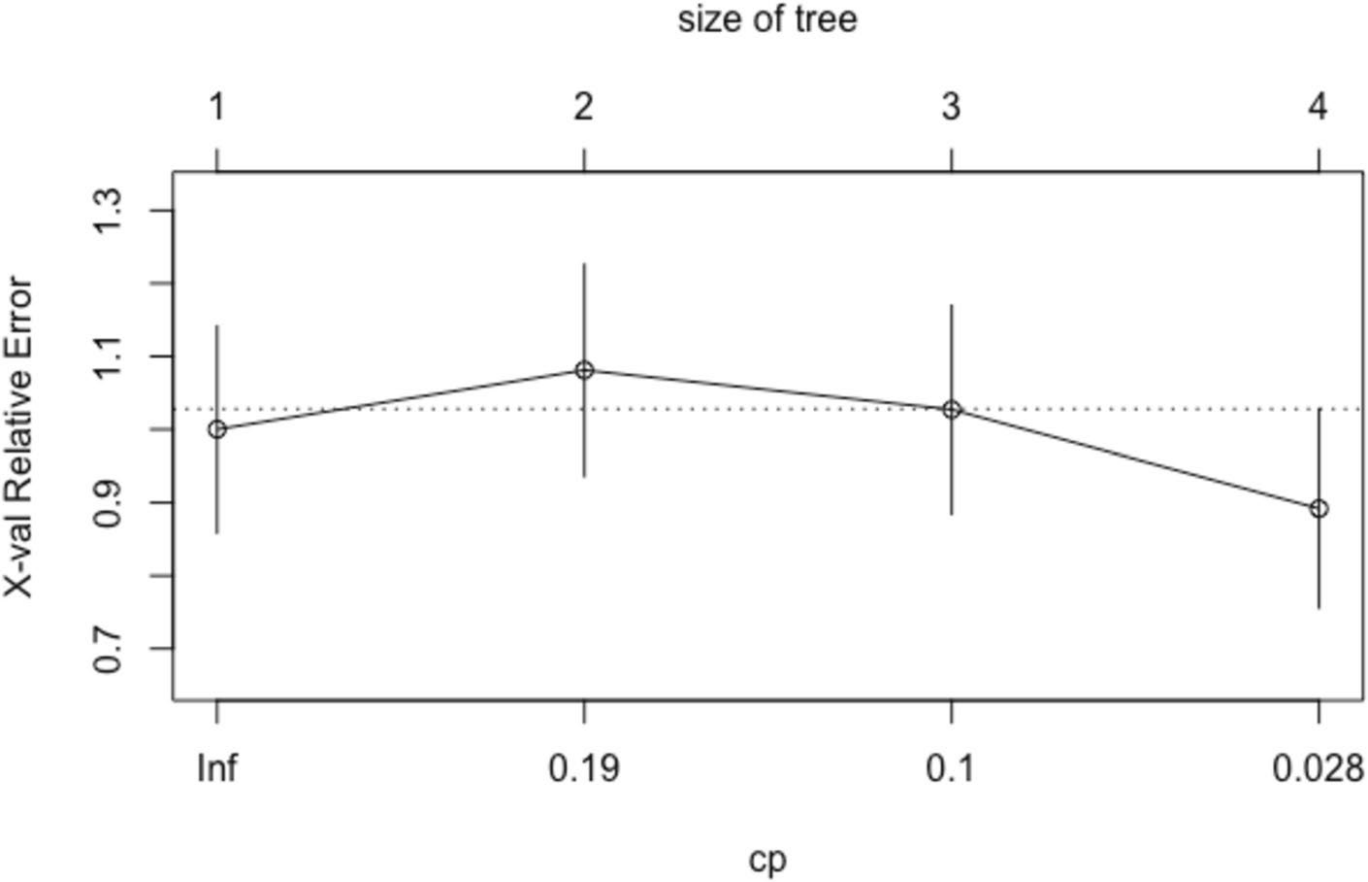
Relative cross-validation error for each sub-tree

As emerged from the figure and tables, the regular version of the auditory go/no-go, the RAN-shapes, and the auditory entrainment_100bpm_ represented the principal root nodes. The relative cross-validation error for each sub-tree, from smallest to largest, is plotted in Fig. 5. The three is pruned based on the complexity parameters (cp, on the *x*-axis) and on the lowest cross-validation error. The overall performance measures of the CART model were obtained through the adoption of a cross-validation procedure. The CART model showed an overall good level of diagnostic accuracy (= 86%, 95% CI: 0.79–0.91), together with a high level of specificity (= 96%, 95% CI: 0.91–0.99) and a low level of sensitivity (= 60%, 95% CI: 0.42–0.75).

#### Step 4. Comparison between monolinguals and MLC in reading and experimental tasks

In the fourth step of the analysis, the MLC’s performance (*N* = 68) was compared to the one of Italian monolinguals (both poor and good readers; *N* = 142; females = 70, males = 72, age on average = 10.39, SD = 1.67). Firstly, as the SES level was unbalanced between the two groups (*X*^2^_(3)_ = 26.8, *p*-value < .001), we checked whether SES influenced performances on reading or experimental tasks by computing ICC. The results of this preliminary analysis did not show any significant results (see Supplementary Table 3).

The two groups showed similar performances in all reading indices, and they did not differ in any discriminant tasks of the *ReadFree* tool (see Table 7 for results of GLMs testing between-group comparisons on reading speed and accuracy; data distributions in the monolingual Italian sample and in MLC are reported for both reading and experimental tasks in Supplementary Figures 1, 2, 3, 4).

**Table 7:** Group comparisons on reading skills

Task	Effect	Mono (*N*)	MLC (*N*)	Model	Estimate	SE	*t*	DF	*p*-value	*p*-value (FDR)	*η*^2^	*R*^2^
Word reading speed (syll./secs.)	Group	142	68	Linear	0.07	0.14	0.54	207	0.58	1	0.01	0.3*
	Age				0.38	0.04	9.25	207	<.001	<.001	0.28	
Pseudoword reading speed (syll./secs.)	Group	142	68	Linear	−0.06	0.09	−0.63	207	0.52	1	0.001	0.26*
	Age				0.23	0.02	8.5	207	<.001	<.001	0.26	
Text reading speed (syll./secs.)	Group	140^#^ˆ	67ˆ	Linear	0.24	0.16	1.53	204	0.12	0.66	0.007	0.4*
	Age				0.51	0.04	11.37	204	<.001	<.001	0.4	
Word reading accuracy (%)	Group	142	68	Tobit	0.77	0.59	1.29	416	0.19	0.91	--	0.06
	Age				0.64	0.16	3.78	416	<.001*	<.001		
Pseudoword reading accuracy (%)	Group	142	68	Tobit	1.13	1.44	0.78	416	0.43	1	--	0.008
	Age				0.72	0.4	1.76	416	0.07	0.57		
Text reading accuracy (%)	Group	142	68	Tobit	34	0.38	0.9	416	0.36	1	--	0.005
	Age				0.17	0.1	1.65	416	0.09	0.6		
Auditory free tapping_100bpm_	Group	140^#^^	64^#^^	Linear	−0.84	5.46	−0.15	201	0.87	1	0.001	0.01
	Age				−2.77	1.5	−1.84	201	0.06	0.6	0.017	
RAN-shapes	Group	142	68	Linear	−0.71	2.77	−0.25	207	0.79	1	0.004	0.31*
	Age				7.46	0.77	9.65	207	<.001	<.001	0.309	
Auditory go/no-go (irregular)	Group	141^^^	68	Linear	−0.10	0.11	−0.86	206	0.38	1	0.000	0.16*
	Age				0.29	0.03	6.33	206	<.001	<.001	0.163	
Auditory go/no-go (regular)	Group	141^^^	68	Linear	−0.13	0.12	−1.11	206	0.26	1	0.001	0.09*
	Age				0.16	0.03	4.66	206	<.001	<.001	0.09	
Visual Rts	Group	142	68	Non-param.	−0.003	0.008	−0.39		0.69	1		0.19*
	Age				−0.016	0.002	−7.08		<.001	<.001		
Auditory entrainment_100bpm_	Group	140^#^^	65^#^^	Non-param.	−1.68	2.6	−0.64		0.51	1		0.004
	Age				0.61	0.72	0.83		0.4	1		
Auditory free tapping_80bpm_	Group	141^#^	67^#^	Non-param.	39.14	23.45	1.66		0.09	0.69		0.03
	Age				10.9	6.48	168		0.09	0.69		
Cocktail party	Group	140^#^	66^#^	Non-param.	−0.11	0.17	−0.67		0.49	1		0.07
	Age				0.18	0.04	3.85		<.001	0.002		
Visual go/no-go (irregular)	Group	140^#^^	68	Non-param.	−0.15	0.09	−1.69		0.09	0.69		0.05
	Age				0.07	0.02	2.94		0.003	0.04		
Visual go/no-go (regular)	Group	140^#^^	68	Non-param.	−0.07	0.09	−0.75		0.45	1		0.01
	Age				0.03	0.02	1.46		0.14	0.94		

^*^F value associated to the *R*^2^ is significant

In line with these results, we concluded that our screening is suitable for all participants regardless of their linguistic knowledge as the *ReadFree* tool did not penalise, on average, MLC students.

#### Step 5. Identification of MLC at risk of dyslexia

As a final step, the set of rules extracted from the CART model run on monolinguals (GR, PR) at step 3 was applied to classify the MLC participants (see Table 8 for the confusion matrix including the classification of good and poor readers made through CART model and the clinical tests).

**Table 8:** Confusion matrix

		Clinical Test		
		*MLC-PR*	*MLC-GR*	
CART model	*MLC-PR*	5	5	10
	*MLC-GR*	11	47	58
		16	52	68

Accordingly, 10 MLC participants were classified as poor readers (MLC-PR) and 58 as good readers (MLC-GR). On the contrary, when clinical criteria based on standard reading tests were applied, 16 MLC were classified as MLC-PR and 52 as MLC-GR. It is noteworthy that only 5 participants classified by the CART as PR corresponded to those identified with clinical criteria (see the confusion matrix in Table 8): the model, thus, returned the 7.3% of false positive rates and the 16.1% of false negative rates, when compared to the clinical classification.

Accordingly, the model showed a low level of sensitivity (= 31%, CI 95%: 0.11–0.59) and a low positive predictive value (= 50%, CI 95%: 0.19–0.81), together with a high level of specificity (= 90%, CI 95%: 0.79–0.97) and high level of negative predictive value (= 81%, CI 95%: 0.69–0.90). The overall accuracy was equal to 76%. Profiles of the 10 MLC participants classified as MLC-PR according to the CART model are reported in Supplementary Table 4. In Supplementary Table 5, we reported the demographic information, parents’ nationality, and the severity of reading deficit (i.e., the number of reading parameters < −1.5 ds) for the MLC that underperformed the standardised clinical reading tests.

## Discussion

As shown by univariate analyses, 6 tasks out of the 12 included in the original version of the *ReadFree* tool could discriminate between GR and PR. More specifically, visual RTs, auditory entrainment, and free tapping (all conditions except for entrainment_80bpm_), cocktail party, RAN-shape, auditory go/no-go (both the irregular and regular versions), and visual go/no-go (both the irregular and regular versions) tasks were considered as cognitive markers of reading difficulties in Italian-monolingual students. The CART model applied to the monolinguals accurately identified the most part of participants as good and poor readers (overall accuracy = 86%, CI 95%: 0.79–0.91) and, in particular, it correctly classified as good readers the most part of those monolinguals who resulted as good readers in standardised reading tests (specificity = 0.96, CI 95%: 0.91–0.99). Accordingly, the *ReadFree* tool can be considered a promising toolbox for the massive screening of Italian-monolingual students aged between 8 and 13 years, even though we are aware that these indices vary depending on sample numerosity and prevalence of the condition in the sample (see Eusebi, 2013).

These preliminary results set the rationale to “refine” the pool of tests to be included in the *ReadFree* tool and to assess, in a future study, their test–retest reliability and their normative clinical data.

As a matter of fact, the results of the decision tree depicted in Fig. 3 give some intriguing clues on the neuropsychological description of reading difficulties in children.

The tree’s main root, namely the task with the highest discriminant value, is associated with fluid transversal functions, i.e., executive functions as measured by the auditory go/no-go (regular) task. This variable represents the ability to control motor behaviour while extracting regularities (or rhythmic information) from auditory input. Interestingly, this first variable gives rise to two distinct branches: (i) a branch that includes the RAN-shapes task, principally related to automation and integration aspects and (ii) a branch that includes the auditory entrainment in the faster version (100 bpm), associated with the timing component. Thus, our study will be discussed by considering these three cognitive levels and by looking at the relationship between executive functions, automation, auditory timing skills, and the reading process.

### Poor reading as a deficit in executive functions, attentional processes, and automation

As mentioned above, the first root node in the decision tree corresponded to the regular version of the auditory go/no-go task; namely, the go/no-go task in which stimuli are delivered with a rhythmic interval. This result is particularly relevant because it suggests that managing regularity and inhibition was highly demanding for the PR group. Indeed, this version of the task requires (i) to update a continuous auditory flow of information, (ii) to process the timing organisation of the stimuli, (iii) to discriminate between the “Go” and the “No-Go” auditory signals. Nevertheless, this specific result seems to better reflect the role of executive functions rather than the role of regularity processing itself. Indeed, one mandatory condition for performing the task well is to inhibit the tendency to “Go”, that can be increased due to the regular presentation of stimuli. Accordingly, we suggest that getting ready to react based on a rhythmic pulse, even when not required by the type of stimulus (namely in the “No-Go” condition) can produce a higher number of “False Alarms”. Therefore, due to the regularity, the stimulus’ arrival is predictable (Large & Jones, 1999; Pagliarini et al., 2020) and the tendency to react at a specific time needs to be efficiently inhibited for avoiding an incorrect response. This may suggest that a good rhythmical awareness could represent a disadvantage. In other words, because of the regularity, executive functions would be the cognitive aspect more stressed by this task and, based on our results, the most powerful language-independent marker of reading difficulties.

The role of executive functions in reading disorders is further supported, based on univariate analyses, by the fact that also the irregular go/no-go task discriminated between monolingual good and poor readers, both in the auditory and visual modality. The crucial role of executive functions, and particularly inhibition, in reading and dyslexia has been deeply explored in the last 20 years (see the review by Farah et al., 2021), in children (e.g., Doyle et al., 2018; Moura et al., 2014; Reiter et al., 2005; Varvara et al., 2014), as well as in adults (e.g., Brosnan et al., 2002; Smith-Spark et al., 2016). For example, Varvara et al. (2014) suggested that dyslexia is characterised by a global deficit in higher-order domain-general cognitive mechanisms, namely a deficit of executive control regardless of the specific modality of stimuli presentation (visual or auditory). However, one may argue that executive functions impairment is a ubiquitous condition across neurodevelopmental disorders, something that can give clinicians some clue about the presence of a neurodevelopmental condition (a general sign) without representing any specific pathology (Willcutt et al., 2008). This line of reasoning is further supported by the results of the logistic regressions on monolingual children. We found that the visual RT task discriminated between monolingual GR and PR: this result supports both the idea of a deficit in attentional orientation and focusing, as already suggested by Facoetti, Paganoni, and Lorusso (2000a), and the possibility of a deficit at the lower cognitive level of alerting (Facoetti et al., 2019; Posner & Petersen, 1990). Once again, this would not be specific to reading deficits but would instead represent a generic sign shared with other neurodevelopmental conditions such as ADHD (Mullane et al., 2011). The deficit in executive functions and attentional components would also be domain independent. For example, we found significant differences between monolingual GR and PR also at the cocktail party task, i.e., in the auditory attentional domain. The performance at this task implies, by definition, the inhibition of auditorily-presented noise distractors and auditory selective attention.

It is noteworthy that, if one considers the PCA run on the monolingual GR group, executive functions represent a factor that has an independent contribution with respect to reading and phonological skills (see F4 in Table 4). This factor included both inhibition tasks, as the go/no-go in all its versions and modalities, and the selective attention measured through the cocktail party task.

Taken together, the results of the decision tree’s root, those of univariate analyses (i.e., the logistic regressions) and of the PCA seem to support the idea that a large part of the poor readers might be characterized by poor executive functions and attentional deficits in both the visual and auditory domain.

However, more specific cognitive signs of reading difficulties emerge if one climbs the decision tree from this general root (i.e., executive functions and attention). In particular, the second node of the decision tree is represented by the performance at the RAN-shapes. Here, it is noteworthy that children who performed worse in the auditory go/no-go, must have also shown low naming skills at RAN to be classified as poor readers according to the results of our CART model (Fig. 3). This result is in line with many studies suggesting that RAN skills are one of the most reliable markers of dyslexia across ages and orthographies (see Araújo & Faísca, 2019; Carioti et al., 2021; Norton & Wolf, 2012 for reviews). Moreover, by adopting a non-alphanumeric novel version of the task, which minimised the role of the reading system (see Carioti et al., 2022a, b for further details), we were able to expand further the empirical findings that support the double deficit hypothesis (Wolf & Bowers, 1999). Interestingly, the association between the deficit at the auditory go/no-go (regular) and RAN tasks in the left branch of the decision tree suggests that the ability to inhibit concomitant distractors, concerning visual stimulus and lexical labels, might represent the core link in the RAN-reading relationship (Bexkens et al., 2015; Van Reybroeck & De Rom, 2019). An ad hoc created experimental paradigm should better address this hypothesis. Interestingly, the results of our PCA analysis further support the RAN-reading relationship. In particular, monolingual GRs showed a strong relationship between RAN and reading fluency (Table 4, F1), in line with several authors (Lervåg & Hulme, 2009; Papadopoulos et al., 2016, for reviews see Norton & Wolf, 2012 and Araújo & Faísca, 2019). Interestingly, the RAN measure did not contribute to the second factor, namely reading accuracy. These findings support the idea that RAN is more deeply related to reading fluency than to reading accuracy and, thus, to the aspect that more clearly reveals the acquired automation of the reading process.

As a final remark, we would like to stress that the dissociation between accuracy and fluency measures in reading is in line with the results reported in one of our previous studies (Carioti et al., 2019) on a completely different sample of participants.

### The other side of poor reading: a deficit of timing skills

Moving to the right branch of the decision tree, the auditory timing deficit seems to prevail. The ability to tap in entrainment with a metronome (entrainment_100bpm_) was particularly relevant for the classification of those poor readers that can adequately perform the regular auditory version of the go/no-go task. The idea of a deficit in readers with dyslexia in conceiving, organising, and reproducing a simple regular pulsation, i.e., a regular pattern, supports causal theories of dyslexia concerning the perception of rapid spectro-temporal alterations of auditory stimuli (see the RAP theory by Tallal, 1980; see Tallal & Gaab, 2006), as well as temporal awareness of regular strong-weak patterns (see the temporal sampling hypothesis by Goswami et al., 2011; Huss et al., 2011). This can be said also for what concerns the cerebellar theory (Nicolson et al., 2001b), if one considers the rhythmic motor production, and the ensuing implicit learning deficits (Gabay et al., 2015; Kahta & Schiff, 2016; Nigro et al., 2015).

According to these theoretical perspectives, timing skills would underpin phonological awareness and produce a cascade influence on reading (Bishop & Snowling, 2004; Démonet et al., 2004; Gabrieli, 2009; Pagliarini et al., 2020; Peterson & Pennington, 2015; Vellutino et al., 2004). This claim seems further supported by the results of the PCA on monolingual good readers. Indeed, rhythmical tasks such as entrainment and free tapping represented a third independent factor together with nonword repetition, which is the main phonological task included in our cognitive assessment. Our results, thus, suggest that timing skills may contribute to identifying poor readers beyond executive functions and automation deficit.

Since a task that correlated with phonology (the entrainment task) represented an independent decision tree’s root, dissociated to the RAN’s one, our results seem, once again, seem to support the double deficit hypothesis (Wolf & Bowers, 1999).

The fact that different rules, depending on different tasks’ scores, can be applied to recognise a poor reader, would also support the view of the multifactorial deficit account (Peterson & Pennington, 2015; for a critical revision, see Compton, 2021). In this perspective, timing skills might represent another relevant language-independent aspect underlying the reading processes.

### A *ReadFree* tool for the assessment of minority-language children

We started the cross-cultural validation of the *ReadFree* tool by a simple basic assumption: the prevalence of reading deficits due to neurodevelopmental conditions should be similar in an unselected group of monolinguals (i.e., in a sample including both good and poor readers) and in a sample of MLC. We therefore carried out a preliminary between-group comparison and the empirical results showed that, on average, the two groups performed similarly both on the standardised clinical reading tests and on the experimental tasks included in the reduced version of the *ReadFree* tool.

Accordingly, we applied the classification rules extracted from the decision tree trained on the monolinguals to the MLC group. As reported in the results section, in the MLC group, the CART model’s automated classification identified 10 participants out of 68 MLC as PR (i.e., 14.7% of the sample). However, when we instead applied the clinical Italian standard criteria to MLC, 16 children out of 68 showed reading difficulties (i.e., 23.5%), a proportion well above the one identified by the *ReadFree* tool. This result suggests that the adoption of Italian monolinguals’ normative data might introduce a bias if applied on MLC; an issue that has been recently highlighted also in the new “Italian guidelines for the identification of Specific Learning Disorder” (see the Recommendation 7.3, cited in the introduction). This may be also the reason why we obtained a low level of sensitivity in our method comparison (sensitivity = 31%; 95% CI: 0.11–0.59). Here, it is noteworthy that the sensitivity has not been computed against a “gold-standard” measure, rather, against a set of psychometrics measures that, by definition, are referred to a population different from MLC, i.e., to monolingual children. Consequently, the standardised Italian reading tests do not seem to be an adequate gold standard to compute reliable indices of sensitivity and specificity (see Trevethan, 2017 for more details) and, thus, they must be carefully interpreted when considering the classification applied on MLC.

In line with the issue of assessing bilinguals for language and reading skills, recently, in Italy, attempts to develop a battery to assess verbal and non-verbal competencies in bilinguals have been pursued (BaBIL; Contento et al., 2013; Eikerling et al., 2022). Similarly, Marinelli et al. (2020) provided normative data for bilingual students on a vast range of neuropsychological tests, including reading. However, these clinical instruments are not available for large-scale screening assessments in the school. The *ReadFree* tool represents a useful tool to fill this gap of knowledge and practice; it represents an easy to manage tool for teachers and clinicians that can be applied in the school irrespectively by the children’s linguistic background. We could say that it is the first form of an *inclusive screening* tool in Italy. Moreover, its language-independent nature makes it easy to be also transposed in other countries.

### Open issues and future directions

Although our *ReadFree* tool, when applied on MLC, obtained an adequate level of performance expressed in terms of overall accuracy (0.76) and a high level of specificity (0.9), if one looks at the confusion matrix reported in Table 8, some intriguing evidence emerged. First, there are 5 MLC classified as PRs by the CART model that, however, did not show any difficulty in the clinical reading tests. These could be considered “False-positive cases” that emerged due to a failure of the CART model classification or due to some neuropsychological factors that are worth a discussion.

One possible (and optimistic) hypothesis is that our screening tool could identify children that will manifest—but do not already show—a reading deficit. This was likely the case for only one child out of 5, given that this child (20ADF, see Supplementary Table 4) showed a failure in one reading parameter (word reading accuracy, see Supplementary Table 4). Further longitudinal studies will be, in this context, very useful to clarify this issue and, at the same time, to understand whether the *ReadFree* tool can predict future manifestations of reading deficits also in younger children.

Another scenario, probably more adherent to our results, is related to the type of deficit recognised by our tool. Although we developed the *ReadFree* tool to assess reading deficits, the monolingual PRs included in the training dataset were heterogeneous, as they included children with a certified clinical diagnosis and children with subtler reading deficits that did not receive yet a formal diagnosis. This view is further supported by some clinical considerations: the diagnosis more often reported in the monolingual PR group is “General Learning Disorders” (12 out of 22 certified participants). This suggests that children included in our sample reported a reading deficit in comorbidity with other learning disorders. Even though this may represent a methodological limitation of our study, it represents an advantage from an ecological point of view: the high level of comorbidities and shared cognitive deficit between dyslexia and dyscalculia (Cheng et al., 2018; Peters et al., 2018; Peters et al., 2020), dyslexia and writing disorders (Döhla & Heim, 2015; Ehri, 2000; Richards et al., 2015) and, more in general, dyslexia and other neurodevelopmental disorders is widely documented (for a review, see Hendren et al., 2018). This fact induced the authors of the latest version of the Diagnostic and Statistical Manual of Mental Disorder (DSM-5; American Psychiatric Association, 2013) to group all learning disabilities under the more comprehensive definition of specific learning disorders (SLDs) and, as highlighted by Hendren et al. (2018, p. 4), “the subtypes of SLDs have been viewed from an academic-subject approach”. Accordingly, a second validation study in which the assessment of children with SLDs is coupled with a more comprehensive neuropsychological assessment (including, for example, arithmetic and writing skills) is needed to better address this issue. Moreover, in order to make our *ReadFree* tool a standard screening practice, we would need to test another independent and broader sample to assess the replicability of the decision tree, test–retest reliability, and extract normative data of the ReadFree tool’s final version.

Furthermore, based on diagnostic parameters, sensitivity levels of our screening were poor in both monolinguals (sensitivity = 60%) and MLC (sensitivity = 31%). Thus, regardless of the issue concerning standardised reading tests applied to MLC, our tool seems to be limited in its ability to detect poor readers. In other words, only 60% of children are correctly recognised as poor readers by our tool, while 40% of them will be classified as good readers. Although this would represent a limit of our tool, we think the sensitivity of our screening will be improved by increasing the sample in further studies and by better studying the functional neuropsychological profile of deficits through an extensive neuropsychological battery. Anyway, by looking at the encouraging good levels of specificity (= 90%) and accuracy (= 88%) in monolinguals and considering that the tool has been developed as a first exploratory screening to orient decisions about the need for subsequent clinical evaluations, we believe that the *ReadFree* may constitute a promising tool that could be very useful if adopted in a school setting.

To conclude, auditory regular go/no-go task, RAN-shapes, and auditory entrainment_100bpm_ emerged as good predictors of reading skills, supporting the idea that some specific integrative and inter-related cognitive components concerning executive functions, attention, and timing are at the basis of the reading process, beyond phonological processing. These results are in line with the universal neuropsychological markers highlighted in the recent meta-analytic study by Carioti et al. (2021) and with the multifactorial view of developmental dyslexia (McGrath et al., 2020; Pennington, 2006).

### Supplementary Information

ESM 1 (PDF 1.01 kb)

## Data Availability

Data will be available on request to the corresponding author.

## Abbreviations

MLC: Minority-language children
RAN: Rapid automatized naming
CART: Classification and regression tree
GR: Good readers
PR: Poor readers
PCA: Principal component analysis

## Footnotes

1 Linee Guida sulla Gestione dei Disturbi Specifici dell’Apprendimento, June 2021, published by the Italian National Guidelines System, Rome 20 January 2022. https://www.scuolainforma.it/wp-content/uploads/2022/01/LG-389-AIPO_DSA.pdf.

2 The socio-economical-status (SES) was computed based on the occupation of each child’s parents. Occupations were classified using the International Standard Classification of Occupations (ISCO team, International Labour Office) and coded along 10 areas. Based on this classification, mothers and fathers’ occupation was collapsed in a unique score, resulting in a three-way classification (high-medium-low level of SES). See Supplementary Table 2 for details on the classification.
