# Supplementary material for: The ReadFree tool for the identification of poor readers: a validation study based on a machine learning approach in monolingual and minority-language children
Source: Ann Dyslexia. 2023 Aug 7;73(3):356–92. doi: 10.1007/s11881-023-00287-3 (PMC10522748; doi:10.1007/s11881-023-00287-3)
Supplement: Supplementary file 1 — (PDF 1.01 kb) [file 11881_2023_287_MOESM1_ESM.pdf]

The *ReadFree tool* for the identification of poor readers: a validation study based on a machine learning approach in monolingual and minority-language children.

Supplementary Materials

1. DESCRIPTION OF THE EXPERIMENTAL TASKS **2**

1.1 SIMPLE REACTION TIMES 2

1.2. RHYTHMIC ENTRAINMENT AND FREE TAPPING TASK 2

1.3. TONE AND GREY-SCALE DISCRIMINATION 4

1.4. COCKTAIL PARTY TASK 6

1.5. RAN-SHAPES TASK 7

1.6. GO/NO-GO TASK 8

1.7. ANTICIPATORY TIMING TASK 9

2. SUPPLEMENTARY TABLES **11**

SUPPLEMENTARY TABLE 1. 11

SUPPLEMENTARY TABLE 2. 12

SUPPLEMENTARY TABLE 3. 13

SUPPLEMENTARY TABLE 4. 14

SUPPLEMENTARY TABLE 5 17

3. SUPPLEMENTARY FIGURES **18**

SUPPLEMENTARY FIGURE 1. 18

SUPPLEMENTARY FIGURE 2. 18

SUPPLEMENTARY FIGURE 3. 19

SUPPLEMENTARY FIGURE 4. 19

1. DESCRIPTION OF THE EXPERIMENTAL TASKS

1.1 Simple Reaction Times

During the *Simple Reaction task*, participants were asked to pay attention and click the mouse as fast as they could, every time they heard a tone (auditory channel) or saw a dot (visual channel). In particular, for each channel, the task has been developed as follows:

- ***Auditory Reaction Times.*** After presenting a starting signal (a traffic light that becomes red, yellow, and green in 6 seconds), the participants see a black screen and are asked to click the mouse as soon as they listen to a pure tone. The pure tone (piano timbre) presented is 440 hertz, corresponding to the note "A" in the English music notation. The task is of 10 trials with a random ISI between 2 and 3.5 seconds.
- ***Visual Reaction Time.*** Participants are asked to click the mouse when they see a white dot in the screen's center. The starting signal is the same as the auditory modality, i.e., a traffic light that becomes red, yellow, and green (in 6 seconds), and the task was developed to be correspondent to the one in the auditory modality (10 trials with a random ISI between 2 and 3.5 seconds). The white dot seen by participants has a diameter of 20 pixels, and its position can vary a maximum of 20 pixels in each of the four dimensions of the screen (left, right, above, and below), to avoid the illusory perception of the movement of a fixed dot.

The time gap between the presentation of the stimuli and the participant's Reaction Times, expressed in milliseconds, has been taken into account as a Reaction Time (RT) in both versions of the task.

Ten trials were presented for each version of the task, but we cut out the first 2 repetitions to analyse just the trials performed after having achieved a complete comprehension of the task. Missing trials and RTs considered outliers (RT > 1 sec.) were further removed. The median RT was taken into account as a final raw score and considered in both univariate and multivariate analyses to obtain one single score for each participant.

1.2. Rhythmic Entrainment and Free Tapping task

The task, in both perceptual modalities, is organised in three phases:

1) *Training phase*. Participants are asked to listen to a regularly repeated pure tone or to observe the repeated presentation of a dot in the centre of the screen;
2) *Entrainment phase*. Participants are instructed to tap - pressing the finger on the mouse – aligned with the tone listened or of the dot observed;
3) *Free tapping phase*. Participants are instructed to tap regularly, maintaining the same regular rhythm listened or observed in phases 1 and 2, even without listening or seeing the stimulus (see Figure S1).

The same task is repeated two times in each perceptual modality: a slower version of 80 bpm and a faster version of 100 bpm. In particular, for each channel, the task has been developed as follows:

- ***Auditory Modality:*** the three stages of the task are marked by a traffic light: (1) when the light is red, participants are asked to listen to the rhythmic repetition of the tone, (2) when the light becomes yellow, participants are instructed to tap in entrainment with the stimuli and, finally, (3) when the light is red no tones are heard, and each participant must tap, maintaining the rhythm. The tone delivered is the same as the one provided in the Reaction Time task (see paragraph 1), and its duration is 200 msec. Consequently, in the slower version, at 80 beats per minute (bpm), tones are delivered with a time gap of 750 msec. (200 msec of stimulus duration + 550 of silence); in the faster version instead, the one at 100 bpm, tones are delivered with a time gap of 600 msec. (200 msec of stimulus duration + 400 of silence).
- ***Visual Modality:*** the three stages of the task were marked by a traffic light and the color of stimuli delivered. After the (1) red traffic light, a red dot appears regularly at the center of the screen, and participants are instructed to watch the dot. Then, after (2) the yellow traffic light, the dot becomes yellow, and participants are asked to tap according to the dot's presentation. Finally, (3) when the dot disappeares, participants are instructed to maintain a regular tap until the end of the task, even without seeing anything on the screen. All the dots presented are of the same size (20 pixels), and their duration is the same as the tone presentation in the auditory modality: the dot duration in the 80 bpm rhythm is 200 msec + 550 of silence, while in the faster condition at 100 bpm, the dot duration is 200 msec + 400 of silence.

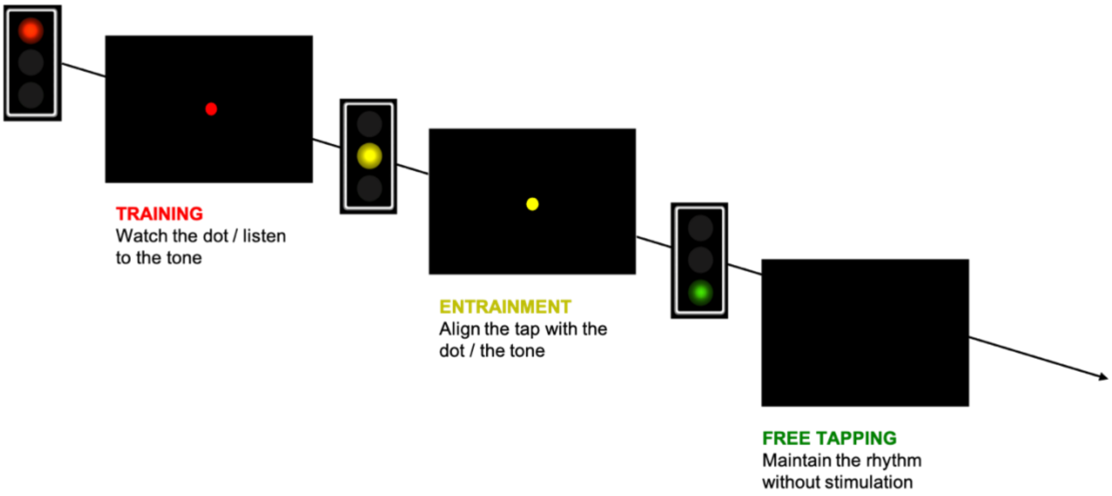

Figure 1| *A representation of the phases of the Entrainment and Free Tapping task in the visual modality.*

In both the auditory and visual modalities, the Entrainment tasks implied 16 repetitions, i.e., 16 beats, while the Free Tapping consists of 12 beats. For data analysis, Entrainment and Free Tapping and the two different rhythmic conditions (80/100 bpm) were considered separately, resulting in 4 analyses per channel. Each participant's between-taps interval (the Tapping Onset – TO) has been recorded; the median of these intervals has been taken into account as a raw score. Moreover, to perform data analysis, this score has been centered in relation to the target rhythm (750/600 msec.) to identify participants who anticipate or delay easily. Variables considered in the analyses are, thus: $Entrainment_{80bpm}$, $Entrainment_{100bpm}$, $Free\text{-}Tapping_{80bpm}$, and $Free\text{-}Tapping_{100bpm}$.

1.3. Tone and Grey-scale discrimination

The task requires discriminating between couples of pure tones or grey squares of similar or different frequency (auditory modality) or intensity (visual modality). Participants were instructed to press two separate buttons on the keyboard to indicate whether the two tones/squares were similar (the "blue button" on the left of the keyboard) or different (the "orange button" on the right of the keyboard; see Figure S2 for an example of visual trial). For each pair of stimuli, the duration was set to 1.5 seconds, with an ISI of 1 second. A new couple of stimuli was always presented immediately after the participant's keypress.

In particular, the two versions of the task were developed as follows:

- ***Tones discrimination task.*** Participants must discriminate between couples of pure tones ranging from 1200 to 0 cents. The "cent" is a logarithmic measure unit designed to count musical intervals without being affected by

frequency contour and, in particular, it is the hundredth part of a semitone (Ellis, 1954). Couples of pure tones presented could be equal (=catch trials) or differ in frequency, resulting in ascending (second tone more acute than the first) or descending (first tone more acute than the second) intervals.

- ***<u>Grey-scale discrimination task.</u>*** Participants must discriminate between two grey squares ranging from 0 and 255 RGB, resulting in a range of grey at a different level of brightness. As in the auditory counterpart, the "similar" couples were made by two squares of the same RGB intensity, while those "different" could be ascending (the second square is brighter than the first) or descending (the first square is brighter than the second). The size of the square is one-tenth of the horizontal screen size.

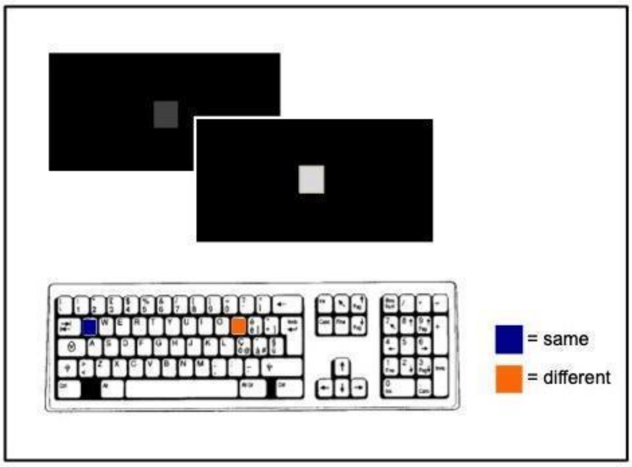

Figure 2| *A representation of the Grey-scale discrimination task.*

Both versions of the task aimed to record the minimum range of variation detectable by each participant based on its discrimination sensitivity. For this reason, an adaptive double staircase procedure (see Viviani & Stucchi, 1989) has been adopted for the selection of Tone/Square sequences and the end of the task. In this adaptive procedure, couples of ascendant and descendent stimuli were selected based on individual performances starting with a wider interval (220 cents/200 RGB) and decreasing it as long as the participant's discriminant skills were accurate. For every inaccurate trial on ascendant or descendent couples, the program made an inversion returning to deliver intervals wider than the last one of 5 units and decreasing the range again after an accurate response. At the third inaccurate response for the same ascendant or descendent interval, i.e., after three inversions, the task ends.

In line with this procedure, the minimum range of detectable between-stimuli variation, namely the Discrimination Threshold (DT) of each participant, is defined by the range detected at least two times in ascendant and descendent pairs. Nevertheless, this adaptive procedure did not fit well with children, and, thus, the discrimination threshold taken as a row score in the current study is represented by the minimum interval accurately detected at least two times, regardless of its ascendant or descendent nature.

1.4. Cocktail Party task

This task, developed only in the auditory modality, requires participants to recognize a target sound in the noise by focusing their attention (top-down selective process).

The task represents the attempt to reproduce one of the most famous attentional effects, defined by experimental psychology with the colloquial term “cocktail-party problem” (Cherry, 1953). In our *Cocktail Party Task,* the target stimulus is not confounded in the noise of a speaking crowd, as in the famous phenomenon, rather the noise is made by multiple sounds selected in the human voice spectrum (between 5 and 1200 cents) to reproduce the effect of a crowd. Confounding sounds are pure tones of different timbres and different frequency intonation; in particular, the number of intonations, i.e., the different “voices” in the crowd, varies between trials. After a training phase that implies the 5-time presentation of the target stimulus, the participant was instructed to search for the target sound in the noise auditorily and to click the mouse when he/she detects it, in three different trials:

- a 3-voice noise trial;
- a 10-voice noise trial;
- a 5-voice noise trial.

Each trail is marked by the presentation of a yellow traffic light of 1-second duration (see Figure S3 for a summary of the task’s phases). 15 (1/3rd) target stimuli in the range of 390-495 cents are confounded between the distractors in each trial. Each stimulus lasts for a total of 1500 msec, considering 650 msec. of fade-in + 250msec of sound + 650 msec. of fade-out. The average duration of a trial is 36.5 seconds.

All the trials are considered together in a single score obtained with the *d-prime* calculation, after the count of "hit", "miss", "false alarm", and “correct rejections” (Green & Swets, 1966).

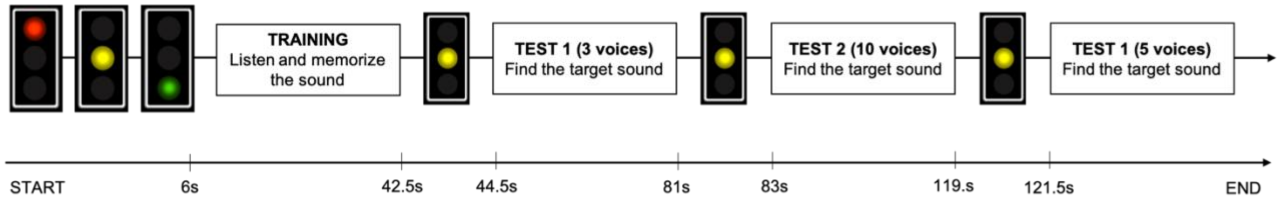

Figure 3| *A representation of phases and average duration of the Cocktail Party effect.*

1.5. RAN-Shapes task

In this renewed version of the Rapid Automatized Naming Task (Denckla & Rudell, 1976), participants had to name as rapidly as possible five standard shapes (heart-circle-triangle-square-star) repeated in grids of different amount of rows and columns (squared matrices). The task is preceded by a training trial in which single shapes are presented separately, and participants are asked to name them. This has been done to check whether the participants have correctly learned the task and every shape's name. Shapes of RAN were inscribed into a 200*200 mm matrix, and the distance between each shape is 4 pixels. The task is composed of 3 different matrices of the same dimension that vary for the number of shapes inscribed in, and/or for perceptual properties (i.e., the texture in the background).

In particular, our RAN-Shapes task consists of three trials:

- the RAN matrix 1 is a 7*7 grid with a total of 49 shapes;
- the RAN matrix 2 consists of a 10*10 grid of the same size as others, in pixels, but with 100 total shapes inscribed. The stimuli are, thus, smaller and closer to each other;
- the RAN matrix 3 is a 7*7 grid equal to the first for size and the number of stimuli (49 in total), but with a background visual interference, conceived to test the sensitivity to crowding effect and/or perceptual interference (see Figure S4).

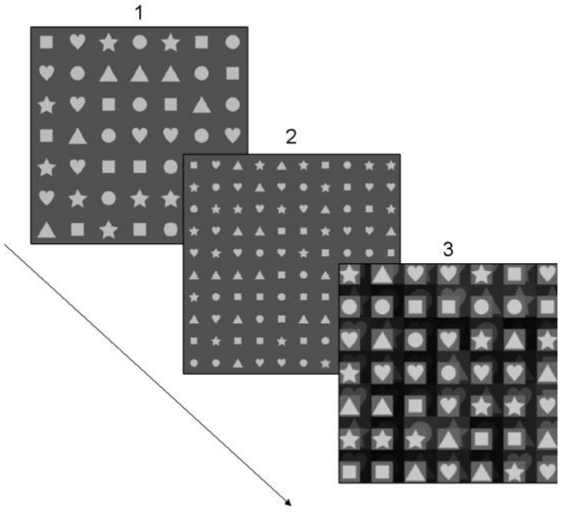

Figure 4| *A representation of the RAN-Shapes task, starting from the 7*7 matrix (1), passing through the more demanding 10*10 matrix (2), and ending with the 7*7 matrix with backward visual interference (3).*

The three matrices were developed to present 2 different types of difficulty and to test (i) whether a higher demand, in terms of the number of stimuli to name and attentional burden can affect naming speed and (ii) whether automation can occur while performing the 3 trials of the task. In particular, the third matrix has been developed to test the crowding effect in susceptibility in developmental dyslexia (see Gori & Facoetti, 2015 for a review). The order of administration of the matrices was always the same for all participants.

In contrast with the classic version of Denckla and Rudel (1976), and with all the posteriors (see Araújo & Faísca, 2019; Norton & Wolf, 2012 for reviews), some shapes are sequentially repeated in our RAN, to test whether the need to articulate a similar or, as in this case, the same phonological output would interfere with the naming speed (as suggested by Jones et al., 2013). Moreover, we decided to record the naming speed in terms of accurately named shapes in 30 seconds, instead of using the traditional methodology, to provide a quick and soft administration, with the advantage to avoid also a strain effect on participants. The effectiveness of this "timed" measure can be considered equal to the untimed one, as emerged in the study by Carioti et al. (2021).

1.6. Go/No-go task

The Go/No-go task was developed in both the auditory and visual channels with the same organization and duration.

The task is widely used in experimental psychology to assess executive functioning and, in particular, has been proposed in the context of electrophysiology as a reliable correlate of inhibition and motor control (Roberts et al., 1994).

As in its classic form, the task requires clicking the mouse when a stimulus learned as a "Go" is presented and not clicking when a "No-Go" stimulus is delivered. In both channels, participants completed two versions of the task:

- an "irregular" version in which jittered ISI could vary between 250 and 1000 msec. of duration
- a "regular" version in which a constant ISI of 850 msec. was maintained. In this last version, the presentation of stimuli can be considered a rhythmic one, and thus the participants were allowed to predict the arrival of a new stimulus at a rhythm of 60 bpm, being ready to choose whether to click or not based on the type of stimulus.

The decision to develop these two versions was due to investigating whether a predictable or unpredictable stimulus presentation can challenge the task. In particular, the two versions were developed in the two channels as follows:

- **Auditory Go/No-go.** Participants were instructed to click when a lower pitch sound was heard (the *Go* sound, 440 Hz frequency, corresponding to the Italian tone "La" - "A" in the English notation) and not click when the higher pitch sound (the *No-Go* sound, 880 Hz frequency, corresponding to the same *Go* note, but higher of an octave) was delivered. After a training phase in which children were instructed to recognize the *Go* sound, marked with the previous presentation of a green traffic light, and the *No-Go* sound, marked with the prior presentation of a red traffic light, each participant completed the first irregular version and then, after a pause, the second regular one.
- **Visual Go/No-go.** Participants were instructed to click when a grey dot appeared at the centre of the screen (the *Go* stimulus) and not click when a yellow dot (the *No-Go* stimulus) was delivered. Each dot was of a diameter of 20 pixels, and its position could vary of maximum 20 pixels in each one of the four dimensions of the screen (left, right, above, and below) to avoid the illusory perception of the movement of a fixed dot. After a training phase in which children were instructed to recognize the *Go* stimulus, marked with the previous presentation of a green traffic light, and the *No-Go* stimulus, marked with the prior presentation of a red traffic light, each participant completed the first irregular version and then, after a pause, the second regular version.

1/3rd of stimuli delivered in both versions of the task were target stimuli (i.e., "Go" signals). For both channels, the scores of the irregular and regular versions were recorded separately by the d-prime based on the number of "hit" and "false alarms" reported by each participant. The measures analyzed were, thus, 2 for the auditory channel and 2 for the visual one and are reported as $dprime_{irregular}$ and $dprime_{regular}$.

1.7. Anticipatory Timing task

The task is an adaptation of the Warning-Imperative Paradigm (Walter, 1964). The auditory version has already been tested on both adults (Pagliarini, 2016; Pagliarini et al., 2020) and children (Pagliarini, 2016; Pagliarini et al., 2020; Persici et al., 2019), while the visual one was *ad hoc* built to the aim of the present study.

In both visual and auditory versions, the task aimed to assess the anticipatory ability, i.e., the ability to generate a timing prediction in relation to a rhythm and to, consequently, properly prepare a motor action.

In particular, in an initial training phase, children were asked to listen to or observe a regularly delivered tone/white dot. The same regular sequence was heard/seen in the testing phase, in which children were instructed to predict the random

arrival of a couple of adjacent tones/bigger dots and click the mouse on the second of them (see Figure S5). In this context, the first stimulus was considered the “Warning”, while the second was considered the “Imperative”.

- **Auditory Anticipatory Timing.** Here the child familiarizes, in the training phase, with a regular beat (a plain metronome) delivered at 80 bpm; during the training phase, the child has been informed that occasionally a couple of different tones will be presented and that he is asked to click when the second one is heard. The beats presented in couples were 440 Hz pure tones (to the note “A” in the English music notation, delivered with a piano timbre) with 8 ms rise and fall times and 200 ms steady-state duration. The total duration of the testing phase was 6 seconds.
- **Visual Anticipatory Timing.** Here the child familiarizes, in the training phase, with the regular appearance of a dot (20 pixels radius) delivered at 80 bpm; during the training phase, the child is instructed to click the mouse when the second of a couple of bigger dots (40 pixels radius) appears. The bigger dots randomly appear with 8 ms rise and fall times and 200 ms steady-state duration. The total duration of the testing phase was 6 seconds.

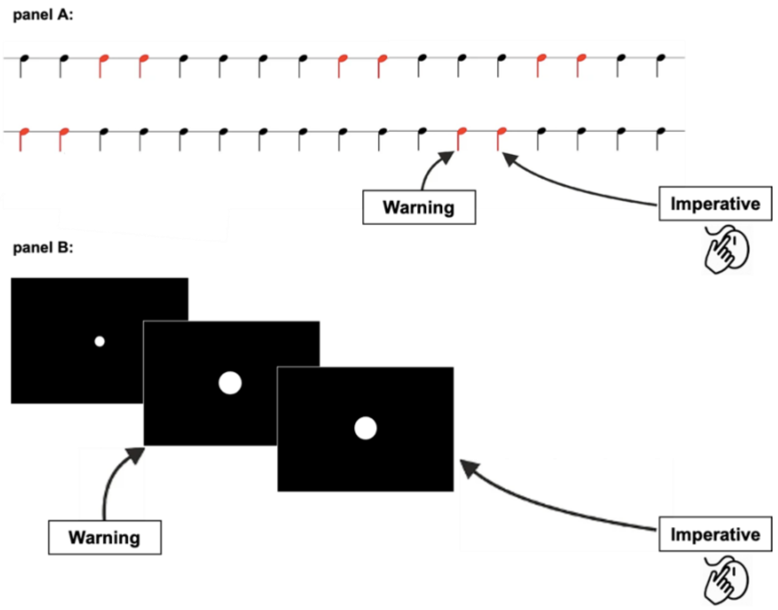

Figure 5| *A representation of the Anticipatory Timing task in both the auditory (panel A) and the visual modality (panel B).*

2. Supplementary Tables

SUPPLEMENTARY TABLE 1| *Parental source country and prevalence in our MLC population (%)*

**Father's Nationality**		**Mother's Nationality**	
Country	*%*	*Country*	*%*
Albania	43.18	Albania	41.86
Ecuador	15.91	Ecuador	16.28
Italy	9.09	Morocco	6.98
China	4.55	Moldavia	6.98
Moldavia	4.55	China	4.65
Peru	4.55	Peru	4.65
Ukraine	4.55	Romania	4.65
Azerbaijan	2.27	Colombia	2.33
Morocco	2.27	Italy	2.33
Portugal	2.27	Germany	2.33
Romania	2.27	Saint-Domingue	2.33
Saint-Domingue	2.27	Spain	2.33
Spain	2.27	Ukraine	2.33
Total	100		100

SUPPLEMENTARY TABLE 2| *International Standard Classification of Occupations and levels of SES extracted on its bases.*

Occupation	Classification	SES-Level
Managers	1	High
Professionals	2	High
Technicians and Associate Professionals	3	Medium
Clerical Support Workers	4	Medium
Services and Sales Workers	5	Medium
Armed Forces Occupations	0	Medium
Skilled Agricultural, Forestry and Fishery Workers	6	Low
Craft and Related Trades Workers	7	Low
Plant and Machine Operators and Assemblers	8	Low
Elementary Occupations	9	Low

SUPPLEMENTARY TABLE 3| *Intraclass Correlation Coefficient (ICC) between Socio-Economical-Status (SES;high-medium-low levels), reading, and experimental tasks.*

**Tasks**	**ICC**	**Lower CI**	**Upper CI**	**N**	**k**	**var w**	**var a**
Word Reading (syll./sec.)	0.00	-0.01	0.40	3	63.91	1.41	0.00
Pseudoword Reading (syll./sec.)	0.02	0.00	0.61	3	63.91	0.58	0.01
Text Reading (syll./sec.)	0.01	-0.01	0.45	3	63.68	2.05	0.01
Word Reading (% acc.)	-0.01	-0.01	0.26	3	63.91	13.58	-0.09
Pseudoword Reading (% acc.)	0.01	-0.01	0.46	3	63.91	80.73	0.52
Text Reading (% acc.)	-0.01	-0.01	0.16	3	63.91	6.48	-0.07
Visual Rts	0.01	-0.01	0.46	3	63.91	0.01	0.00
Auditory Entrainment100bpm	0.00	-0.01	0.37	3	63.45	1925.37	-0.61
Auditory Free Tapping80bpm	-0.01	-0.01	0.24	3	63.45	20875.70	-156.53
Auditory Free Tapping100bpm	0.00	-0.01	0.33	3	63.45	2670.98	-7.50
Cocktail Party	-0.02	-0.02	0.02	3	62.52	1.27	-0.02
RAN-Shapes	0.02	-0.01	0.56	3	63.91	491.65	8.13
Auditory Go/No-Go (Irregular)	0.01	-0.01	0.47	3	63.91	0.75	0.01
Auditory Go/No-Go (Regular)	0.01	-0.01	0.47	3	63.91	0.84	0.01
Visual Go/No-Go (Irregular)	0.01	-0.01	0.47	3	63.38	0.38	0.00
Visual Go/No-Go (Regular)	0.03	0.00	0.63	3	63.38	0.40	0.01

SUPPLEMENTARY TABLE 4| *Cognitive profiles of MLC that were classified as Poor Readers (PR) by the prediction of the CART model. Clinical scores reported in the cognitive assessment are reported together with performances in the ReadFree Screening Tool's tasks. More severe deficits in reading tasks are highlighted in dark grey, while moderate deficits are highlighted in light grey. Data concerning reading fluency are reported in standard deviations while reading accuracy is reported in percentiles.*

Code	***14SM^***	***30DM^***	***30XH***	***12RS***	***12LW***	***24AA***	***34LT***	***53RMT***	***20ADF***	***09EC***
Nationality	Albania	Albania	China	Albania	Sri Lanka	Albania	Albania	Colombia	Romanian + Italian	Albania
Born in Italy	Yes	Yes	No	Yes	Yes	Yes	Yes	Yes	Yes	Yes
Age (years)	8	10	9	9	10	8	8	9	13	9
Age (month)	105	130	115	108	124	101	99	116	163	111
Grade	3rd	5th	4th	4th	4th	3rd	3rd	4th	8th	4th
Gender	F	M	M	M	M	M	M	F	F	M
Handedness (R/L)	R	R	R	R	R	R	R	R	R	R
Non-verbal Reasoning (Standard Score)	88	63	100	97	88	94	74	97	52	97
Digit FW (Span)	0.7	-1.54*	-1.27	0.54	-0.63	1.7	-1.3	0.54	-1	-1.27
Digit BW (Span)	2.12	-0.77	-0.6	-0.6	-0.77	0.87	-0.37	1.4	-0.33	0.4
Nonword Repetition (acc. – z scores)	-0.38	0	**-2.69****	-0.77	0.19	-0.17	0.86	0.77	N.A.	-0.19
Word Reading (Total Time – z scores)	**-1.78***	**-7.37****	-0.47	0.21	-0.38	-0.59	0.67	0.14	-1.02	-0.19

Code	14SM^	30DM^	30XH	12RS	12LW	24AA	34LT	53RMT	20ADF	09EC
Word Reading (syll./sec. – z scores)	-1.40	**-2.81****	-0.66	-0.02	-0.6	-0.81	0.51	-0.10	-1.07	-0.42
Word Reading (errors – z scores)	**< 5^{th**}**	**< 10^{th*}**	**< 10^{th*}**	**< 5^{th**}**	**< 10^{th*}**	> 15th	> 15th	> 15th	> 15th	> 15th
Pseudoword Reading (Total Time – z scores)	**-1.84***	**-6.86****	0.02	0.77	-0.31	0.11	0.95	0.72	-0.07	0.04
Pseudoword Reading (syll./sec. – z scores)	-1.46	**-2.34****	-0.21	0.63	-0.48	-0.18	1.07	0.56	-0.32	-0.18
Pseudoword Reading (errors – z scores)	**< 5^{th**}**	**< 5^{th**}**	**< 10^{th*}**	> 15th	> 15th	> 15th	> 15th	> 15th	> 15th	> 15th
Text Reading (syll./sec. – z scores)	-1.45	**-3.53****	**-1.51***	-0.53	-0.3	-1.17	-0.12	-0.10	-1.23	-0.3
Text Reading (errors – z scores)	**< 5^{th**}**	**< 5^{th**}**	**< 10^{th*}**	**< 5^{th**}**	**< 5^{th**}**	> 15th .	> 15th .	> 15th .	> 15th .	**< 10^{th*}**

** below -2 sd/ below 5th percentile; * below -1.5 sd/ below 10th percentile.
ˆThese participants are siblings.

SUPPLEMENTARY TABLE 5| *Demographic information of MLC rated as Poor Readers (PR) based on Italian normative data of reading tests. Age in years (y) and months (m) is reported, together with information about gender, parents' nationality, and the number of deficits observed in Word, Pseudowords, and Text reading for both fluency and accuracy (range of value from 0 to 6). Additional information regarding the degree of exposure to the minority language is also reported.*[1]

ID	Age (y)	Age (m)	Grade	Biological Gender	Handedness	Mother's Nationality	Father's Nationality	Born in Italy	Reading deficits (n)	Degree of exposure to the minority language with the mother (%)	Degree of exposure to the minority language with the father (%)	Degree of exposure to the minority language at home (%)
**36AV**	**8**	**96**	**3rd**	**F**	**Right**	**Ukraine**	**Ukraine**	**yes**	**3**	**16.67**	**8.33**	**16.67**
**14SM***	**8**	**105**	**3rd**	**F**	**Right**	**Albania**	**Albania**	**yes**	**5**	**22.91**	**0**	**22.91**
**12RS**	**9**	**108**	**4th**	**M**	**Right**	**Albania**	**Albania**	**yes**	**2**	**0**	**12.5**	**4.17**
24DIF	9	109	4th	M	Right	Romania	Romania	yes	2	0	0	4.17
**29AB**	**9**	**112**	**4th**	**F**	**Right**	**Albania**	**Albania**	**yes**	**2**	**25**	**12.5**	**25**
32LM	9	112	4th	M	Right	Ecuador	Ecuador	yes	6	29.17	8.33	29.17
10EM	9	115	4th	F	Right	Ecuador	Ecuador	yes	2	0	0	37.5
**30XH**	**9**	**115**	**4th**	**M**	**Right**	**China**	**China**	**no**	**3**	**22.92**	**2.08**	**22.92**
20JB	9	117	4th	M	Right	Albania	Albania	no	3	27.08	20.83	27.08
**16K**	**10**	**120**	**4th**	**F**	**Right**	**Peru**	**Peru**	**no**	**2**	**20.83**	**10.42**	**29.16**
04XZ	10	127	4th	F	Right	China	China	no	6	22.92	6.25	22.92
**12LW**	**10**	**128**	**4th**	**M**	**Right**	**Sri-Lanka**	**Sri-Lanka**	**yes**	**2**	**0**	**0**	**0.5**
**30DM**	**10**	**130**	**5th**	**M**	**Right**	**Albania**	**Albania**	**yes**	**6**	**12.50**	**8.33**	**12.5**
21NSM	11	133	5th	F	Right	Ecuador	Ecuador	yes	2	*0*	*0*	*12.5*
27SK	13	165	8th	M	Right	Italy	Albania	yes	6	*NA*	*NA*	*NA*
**04FG**	**13**	**165**	**8th**	**M**	**Right**	**Albania**	**Albania**	**yes**	**6**	**0**	**0**	**0.5**

[1] The degree of cumulative exposure to the minority language has been assessed using the PLQ Interview (Carioti et al., 2022, September 13). In particular, the three indices reported measured the degree of cumulative exposure in terms of the percentage of hours per day spent actively speaking the minority language with the mother, the percentage of hours per day spent actively speaking the minority language with the father, and the percentage of hours per day spent in passively listening to the minority language in the home context.

3. Supplementary Figures

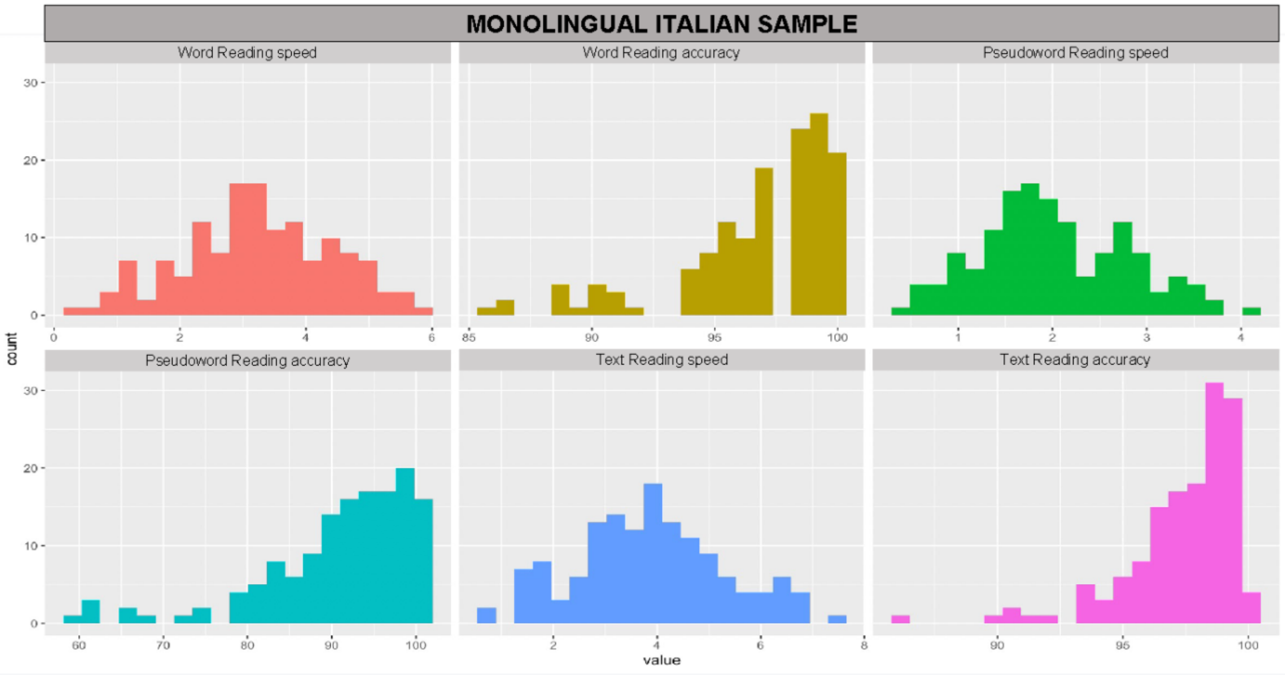

SUPPLEMENTARY FIGURE 1. Data distribution for standardised reading measures in the monolingual Italian sample, including both good and poor readers.

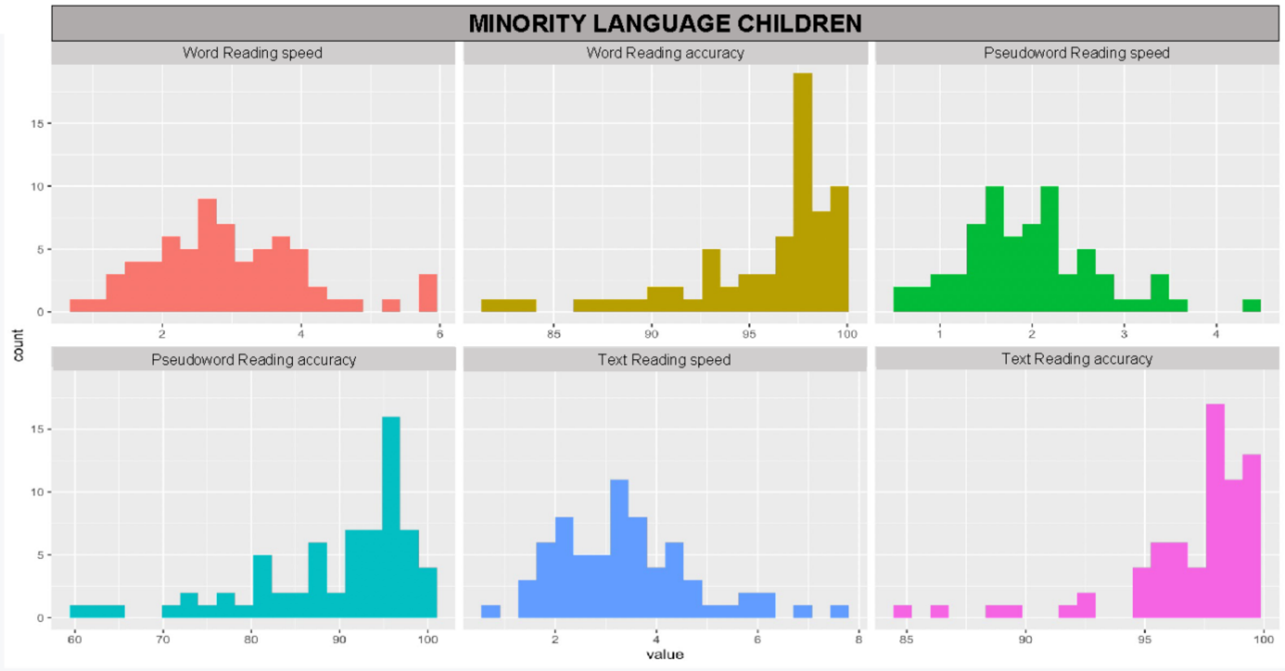

SUPPLEMENTARY FIGURE 2. Data distribution for standardised reading measures in the MLC sample, including both good and poor readers.

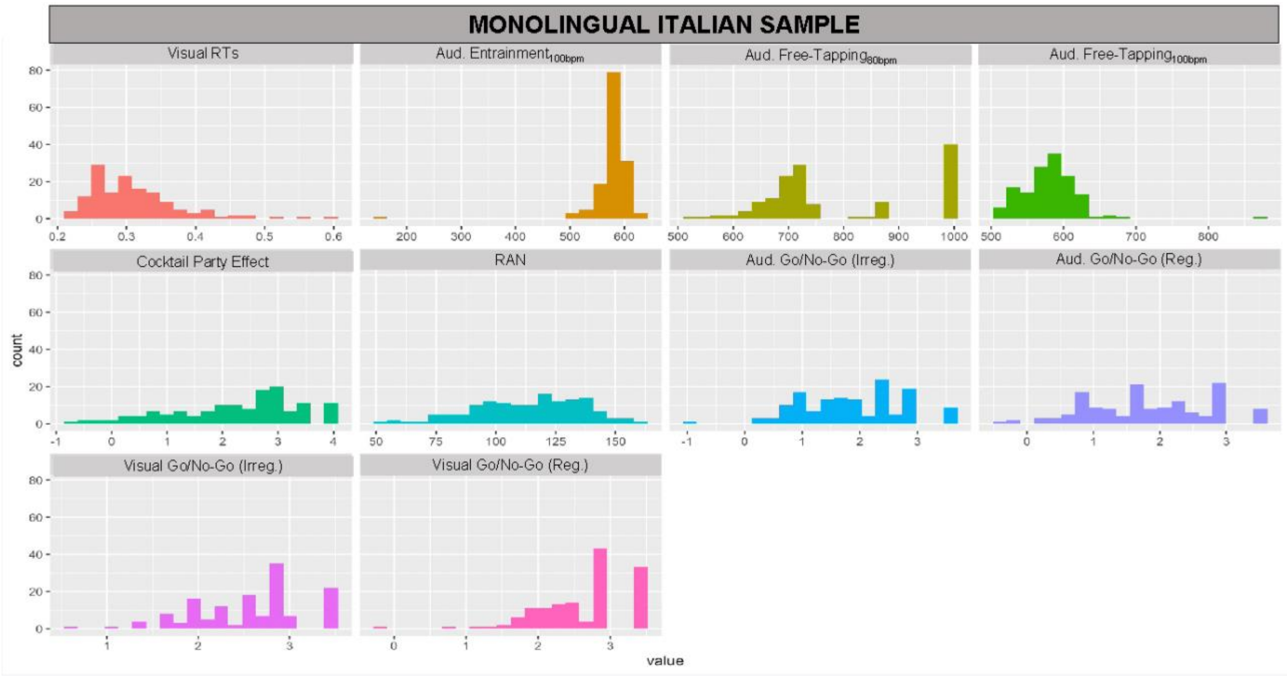

SUPPLEMENTARY FIGURE 3. Data distribution for discriminant tasks of the ReadFree tool in the monolingual Italian sample, including both good and poor readers.

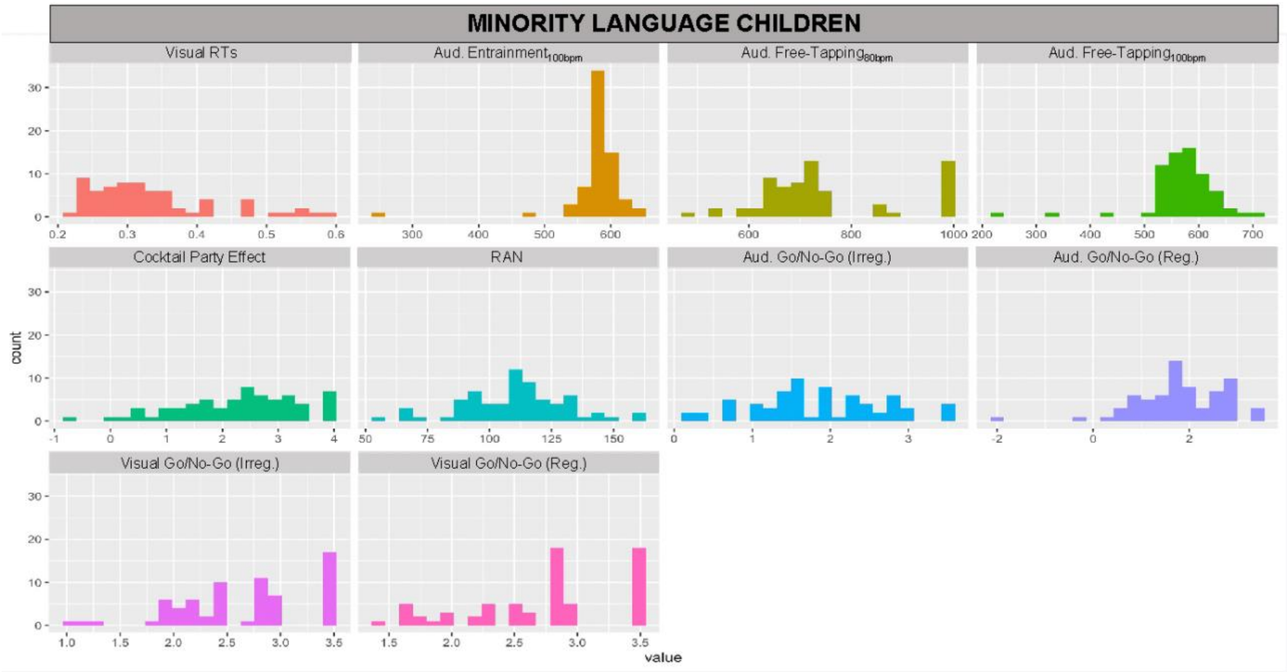

SUPPLEMENTARY FIGURE 4. Data distribution for discriminant tasks of the ReadFree tool in the monolingual Italian sample, including both good and poor readers.
